# Order–Disorder, Symmetry Breaking, and Crystallographic Phase Transition in a Series of Bis(*trans*-thiocyanate)iron(II) Spin Crossover Complexes Based on Tetradentate Ligands Containing 1,2,3-Triazoles

**DOI:** 10.1021/acs.inorgchem.3c00830

**Published:** 2023-05-25

**Authors:** Maksym Seredyuk, Kateryna Znovjyak, Francisco Javier Valverde-Muñoz, M. Carmen Muñoz, Volodymyr M. Amirkhanov, Igor O. Fritsky, José Antonio Real

**Affiliations:** † Instituto de Ciencia Molecular (ICMol)/Departamento de Química Inorgánica, Universidad de Valencia, 46980 Paterna, Valencia, Spain; ‡ Department of Chemistry, Taras Shevchenko National University of Kyiv, 64/13, Volodymyrska Street, 01601 Kyiv, Ukraine; § Departamento de Fisica Aplicada, Universitat Politècnica de València, Camino de Vera s/n, 46022 Valencia, Spain

## Abstract

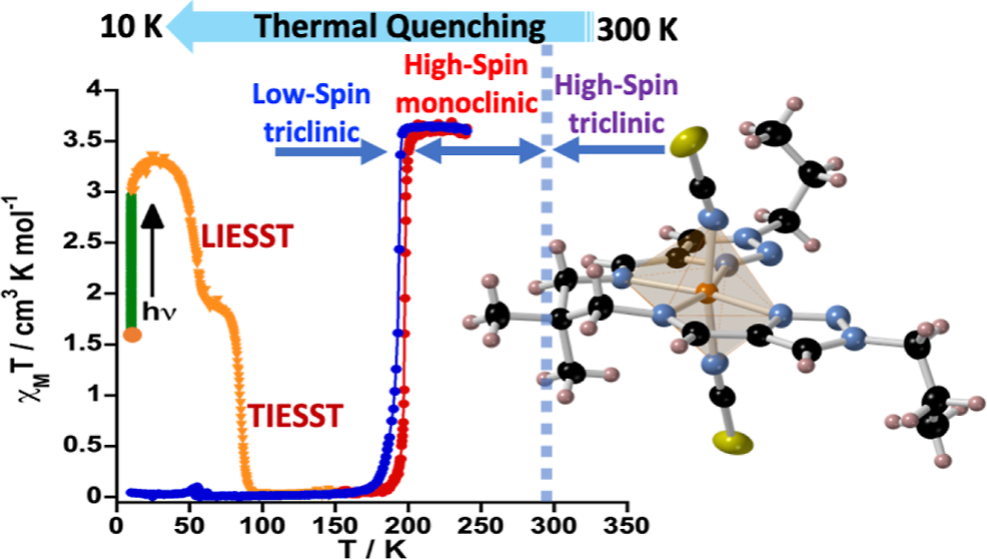

We report herein a series of neutral *trans*-thiocyanate mononuclear spin crossover (SCO) complexes, [Fe^II^L(NCS)_2_] (**1**–**4**), based on tetradentate ligands L obtained by reaction of N-substituted 1,2,3-triazolecarbaldehyde with 1,3-propanediamine or 2,2-dimethyl-1,3-diaminopropane [L = *N*^1^,*N*^3^-bis((1,5-dimethyl-1*H*-1,2,3-triazol-4-yl)methylene)propane-1,3-diamine/-2,2-dimethylpropane-1,3-diamine, **1**/**2** and *N*^1^,*N*^3^-bis((1-ethyl/1-propyl-1*H*-1,2,3-triazol-4-yl)methylene)-2,2-dimethylpropane-1,3-diamine, **3**/**4**]. The thermal-induced SCO behavior is characterized by abrupt transitions with an average critical temperature (Δ*T*_1/2_)/hysteresis loop width (Δ*T*_hyst_) in the range 190–252/5–14 K, while the photo-generated metastable high-spin (HS) phases are characterized by *T*_LIESST_ temperatures in the range 44–59 K. Single crystal analysis shows that except **1**, all compounds experience reversible symmetry breaking coupled with the thermal SCO. Furthermore, **4** experiences an additional phase transition at ca. 290 K responsible for the coexistence of two HS phases quenched at 10 K through LIESST and TIESST effects. The molecules form hexagonally packed arrays sustained by numerous weak CH···S and C···C/S···C/N···C bonds involving polar coordination cores, while non-polar pendant aliphatic substituents are segregated inside, occupying hexagonal channels. Energy framework analysis of complexes with one step SCO transition (**1**, **2,** and **4**) shows a correlation between the cooperativity and the amplitude of changes in the molecule–molecule interactions in the lattice at the SCO transition.

## Abstract 2

Magnetic, photo-magnetic, calorimetric, and crystallographic studies of a series of [Fe^II^L(NCS)_2_] neutral *trans*-thiocyanate mononuclear spin crossover complexes based on 1,2,3-triazole-bearing tetradentate ligands reveals a variety of order−disorder, symmetry breaking, and crystallographic phase-transition events including interesting TIESST and LIESST kinetic effects coupled with cooperative spin-state transitions.

## Introduction

Fe^II^ spin crossover (SCO) complexes are a relevant class of molecular switching materials that have aroused increasing interest during the last seven decades.^1−3^ Their appealing intrinsic properties and synergistic interplay with extrinsic properties have focused much attention on the preparation of multifunctional molecular and hybrid SCO materials with potential application in sensors and memory devices.^4−7^ Typically triggered by the action of temperature, pressure, light, or inclusion of analytes, these complexes switch between the diamagnetic low-spin (LS, t_2g_^6^e_g_^0^) and paramagnetic high-spin (HS, t_2g_^4^e_g_^2^) electronic configurations in a reversible, controllable, and detectable way. Owing to the antibonding nature of the e_g_ orbitals, their population (HS) ↔ depopulation (LS) is strongly coupled with structural changes at the molecular level, which can be transmitted cooperatively across the crystal, conferring bistability (hysteresis) to the magnetic, optical, and dielectric properties of the material.^8−14^ Cooperativity is one of the most significant motivations in the search for new SCO materials, which can be achieved by coupling the Fe^II^ SCO centers through constructive elastic interactions.^15,16^ In the so-called supramolecular approach, the coupling between discrete SCO centers takes place exclusively through elusive intermolecular interactions, usually hydrogen bonding and/or π–π stacking.^17−20^ Alternatively, these interactions can be replaced, in a more or less extension, by robust covalent bonds in the polymeric approach, thereby affording 1–3D coordination frameworks.^21−24^ It is important to note that, despite the differences between both approaches, there is no prevalence of any one of them since very wide thermal hysteresis has been described irrespective of the approach used.^20,25,26^ Independently of the chosen approach, the occurrence of crystallographic phase transition is an additional factor that can be used to trigger and/or tune the SCO.^27−30^

In the supramolecular approach, neutral mononuclear complexes formulated [Fe(L)_*n*_(NCS)_2_] constitute an important source of Fe^II^ SCO behaviors since being L a chelate *N*_2_-didentate (*n* = 2) or *N*_4_-tetradentate (*n* = 1) ligand bearing one or two α-diimine functions, respectively, combined with coordinating SCN^–^ anions usually afford the [FeN_6_] polyhedron with the ligand field strength just right to induce spin state switching. Furthermore, these neutral SCO complexes have also attracted interest because they are amenable to be processed as molecular thin films, thereby affording the possibility to test them in spintronic devices.

**Scheme 1:**
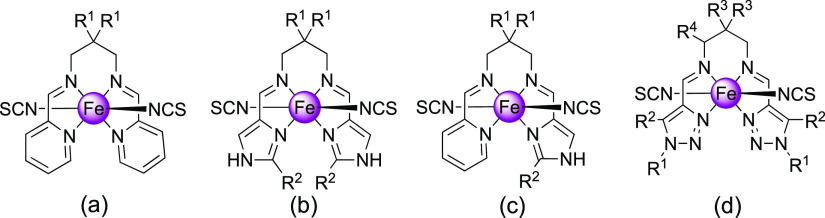
Molecular Structure of [Fe(L)(NCS)_2_] Complexes So Far Investigated Based on Pyridine (a), Imidazole (b), Mixed Pyridine/Imidazole (c), and 1,2,3-Triazole (d) Rings (See Text)

By far, the series of [Fe(L)_2_(NCS)_2_] complexes, containing archetypal examples of SCO behavior, has received much more attention^31^ than the [Fe(L)(NCS)_2_] ones. In the latter, the *N*_4_-tetradentate L ligand typically results from 2:1 Schiff condensation between an appropriate carbaldehyde and 2,2-R-propane-1,3-diamine (R = H, Me). Usually the ligand L saturates the four equatorial sites of the [Fe^II^N_6_] octahedron forcing the SCN^–^ ligands to adopt a trans configuration. As far as we know, first systematic studies on such Fe^II^ complexes were realized by Tuchagues et al. using imidazole- and/or pyridine–carbaldehyde precursors (Scheme 1a–c).^32−34^ More recently, functionalization of the aromatic rings^35^ or the propane diamine fragment^36^ with long aliphatic chains and its effect on the SCO properties has been investigated. In particular, Hagiwara et al. have extended this family of compounds incorporating 1,2,3-triazole rings, and the resulting SCO complexes (Scheme 1d) have afforded interesting examples of polymorphism, breaking symmetry and strong cooperative transitions.^37,38^

**Scheme 2:**
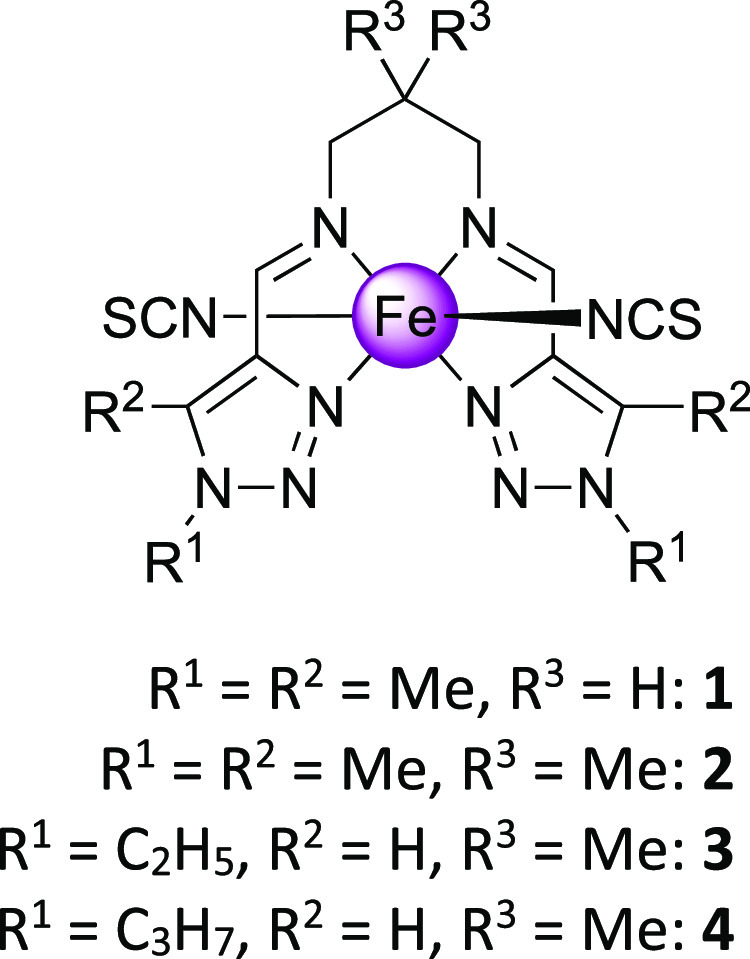
Molecular Structure of [Fe(L)(NCS)_2_] Complexes Reported Here

Aiming at exploring new SCO behaviors in this family of 1,2,3-triazole-based complexes, we have synthesized and characterized four new [Fe(L)(*trans*-NCS)_2_] complexes derived from the condensation of 1,5-dimethyl-, 1-ethyl-, and 1-propyl-1,2,3-triazole-4-carbaldehyde with 2,2-R-propane-1,3-diamine with R = H and Me (complexes **1–4**, Scheme 2). The magnetic, photomagnetic, calorimetric, and structural data of the resulting complexes show the occurrence of cooperative SCO behaviors accompanied by symmetry breaking associated with order–disorder events or crystallographic phase transition.

## Results

### Magnetic Properties

**Figure 1:**
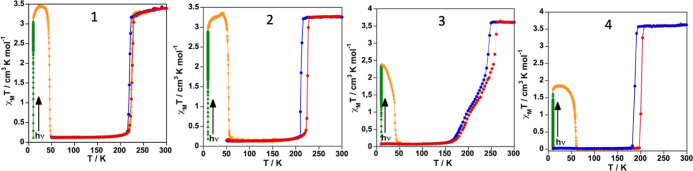
Magnetic and photomagnetic properties of **1–4**. Blue and red dots correspond to the cooling and heating modes, respectively. Green and orange triangles correspond to the irradiation at 10 K of the LS state (LIESST effect) and the heating process at 0.3 K min^–1^, respectively.

The magnetic properties of **1**–**4** recorded at a rate of 1 K min^–1^ represented in the form of χ_M_*T* vs *T* plots are shown in Figure 1 (χ_M_ is the molar magnetic susceptibility, and *T* is the temperature). At 300 K, the χ_M_*T* values were found in the range 3.28–3.63 cm^3^ K mol^–1^, values consistent with the Fe^II^ centers in the HS state. Upon cooling, χ_M_*T* first remains practically constant and then drops to reach values close to zero consistent with a fully populated LS state. The HS → LS switch takes place in one abrupt step for **1**, **2,** and **4** and in two steps for **3,** the first being abrupt and the second much more gradual. In the heating mode, the χ_M_*T* values do not match those of the cooling mode showing the occurrence of hysteretic behavior. The average equilibrium temperatures, *T*_1/2_, at which the HS and LS molar fractions are equal to 0.5 obtained from the cooling-heating modes as well as the hysteresis width, Δ*T*_hyst_, are respectively 221.7/6 K (**1**), 217.8/13.6 K (**2**), 252/14 K (**3**, first step), 200/5 K (**3**, second step), and 194.3/13.5 K (**4**).

Photo-generation of the metastable HS* state from the LS state, the so-called light-induced excited spin state trapping (LIESST) experiment,^39^ was carried out at 10 K irradiating microcrystalline samples of the **1–4** with green light (λ = 532 nm). The four samples undergo the LIESST effect with different yields. Indeed, χ_M_*T* saturates to values ca. 2.9–3.0 cm^3^ K mol^–1^ for **1** and **2**, 2.4 cm^3^ K mol^–1^ for **3** and 1.7 cm^3^ K mol^–1^ for **4**. Subsequently, the light was switched off and the temperature was increased at a rate of 0.3 K min^–1^, inducing a gradual increase of χ_M_*T* that attains a maximum value of 3.40/3.30 cm^3^ K mol^–1^ in the interval of 20–40 K for **1**/**2** and 1.84 cm^3^ K mol^–1^ for **4** in the interval 15–30 K. In contrast, χ_M_*T* decreases continuously from the saturation value ca. 2.34 cm^3^ K mol^–1^ for **3** at 10 K. These values correspond to a HS* population of ca. 100% (**1**, **2**), 66.7% (**3**), and 47% (**4**). The rise in χ_M_*T* observed for **1**, **2,** and **4** reflects the thermal population of different microstates originated from the zero-field splitting of the HS* state. As the temperature increases, χ_M_*T* decreases rapidly until joining the thermal SCO curve at ca. 48 (**1**), 58 (**2**), 44 (**3**), and 64 (**4**) K, indicating that the metastable HS* state has relaxed back to the stable LS state. The corresponding *T*_LIESST_ temperatures, evaluated as δ(χ_M_*T*)/δ*T*,^40^ are respectively ca. 45, 54, 39, and 59 K. These temperatures are consistent with the inverse-energy-gap law, that is, the metastability of the photo-generated HS* species decreases as the stability of the LS increases.^41−43^ The lower HS* population attained in **3** and particularly in **4** can be understood in terms of crystal packing effects derived from crystallographic phase transitions (vide infra).

### Calorimetric Properties

**Figure 2:**
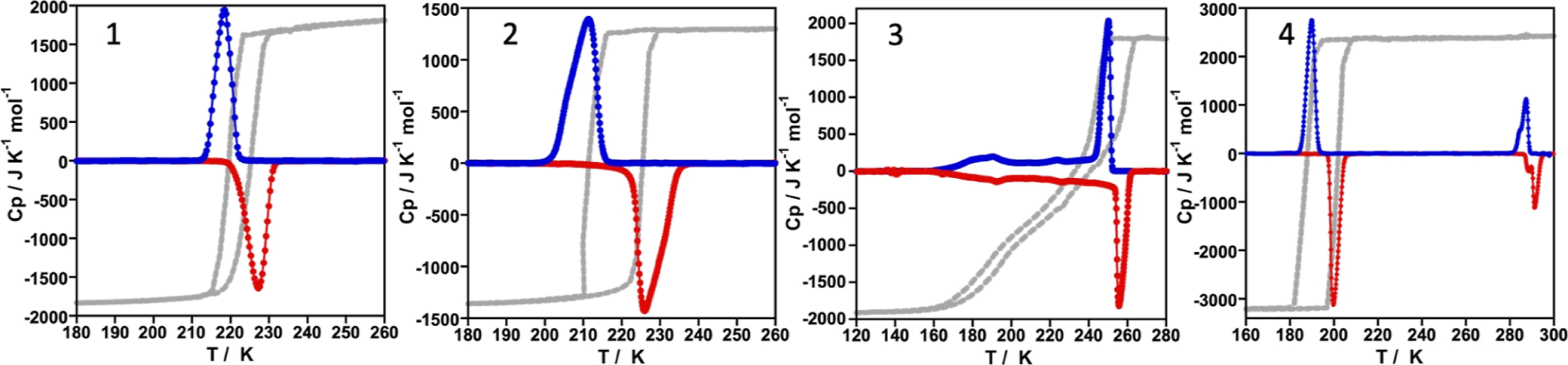
ΔC_*p*_ vs *T* plots for **1–4** in the cooling (blue) and heating (red) modes. Gray lines are the corresponding χ_M_*T* vs *T* plots.

Differential scanning calorimetric (DSC) measurements recorded at 10 K min^–1^ were carried out for **1**–**4** to analyze the thermal dependence of the heat capacity at constant pressure Δ*C*_*p*_ (see Figure 2). The average enthalpy Δ*H* and entropy variations Δ*S* (= Δ*H*/*T*_c_) (being *T*_c_ the temperature at the maximum/minimum of Δ*C*_*p*_ vs *T* plot) associated with the exo- and endo-thermic peaks are, respectively, 9.4 kJ mol^–1^ and 42.2 J K^–1^ mol^–1^ for **1** and 10.6 kJ mol^–1^ and 48.7 J K^–1^ mol^–1^ for **2**, 19.5 kJ mol^–1^ and 83.0 J K^–1^ mol^–1^ for **3,** and 12.6 kJ mol^–1^ and 64.5 J K^–1^ mol^–1^ for **4**. These Δ*H* and Δ*S* values are consistent with the occurrence of more or less cooperative complete SCO. The *T*_c_^av^ obtained from DSC data agree reasonably well with the *T*_1/2_^av^ obtained from magnetic measurements. Interestingly, the Δ*C*_*p*_ vs *T* plot for **4** shows an additional exo- and endo-thermic peak characterized by an average Δ*H*_PT_^av^ = 3.9 kJ/mol and Δ*S*_PT_^av^ = 13.4 J K^–1^ mol^–1^ centered at a *T*_c_^av^ = 289.7 K. It is worth noting that this change in Δ*C*_*p*_ taking place ca. 95 K higher than the SCO apparently seems to be uncoupled with the SCO and has no remarkable consequences on the HS population of **4**.

### Phase Transition and Partial Locking of the Photo-Induced HS* State in **4**

**Figure 3:**
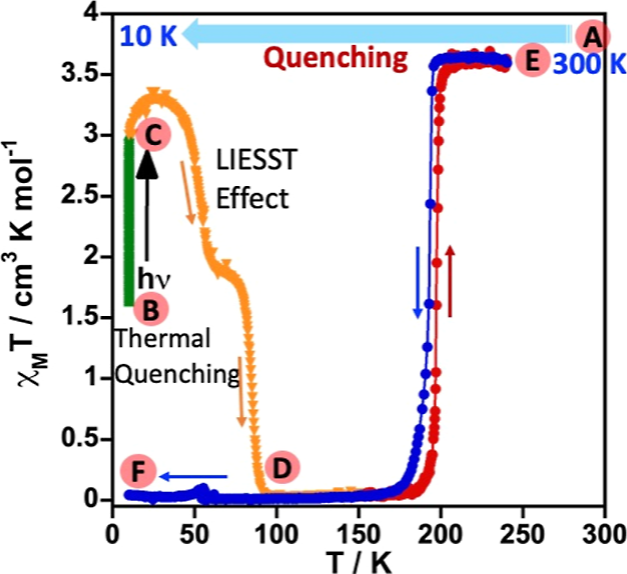
Thermal quenching of **4** from 300 K (A) down to 10 K (B) and LIESST behavior of the remaining LS molecules (C,D). Green and orange triangles correspond to the variation in χ_M_*T* due to irradiation at 10 K and the heating at 0.3 K min^–1^, respectively. Red and blue dots correspond to the heating and cooling modes. The sequence of the experiment was **A** → **B** → **C** → **D** → **E** → **F**.

The exo- and endo-thermic processes centered at 289.7 K observed in **4** can a priori be associated with a crystallographic phase transition originated from the inherent structural instability associated with the aliphatic chains, that is, propyl groups in **4** (vide infra). Taking this into account and based on our previous results,^30^ we decided to investigate the thermal quenching of the HS phase at 300 K (**A**) through fast cooling down to 10 K (**B**), the so-called temperature-induced excited spin state trapping (TIESST) experiment, subsequent LIESST effect of the potentially remaining LS phase (**C**), and the thermally induced relaxation to the fully populated ground LS state (**D**) (see Figure 3). The χ_M_*T* value of the resulting phase at 10 K was 1.61 cm^3^ K mol^–1^, suggesting the thermal trapping of practically 50% of the Fe^II^ centers. This quenched phase was irradiated with green light reaching saturation of χ_M_*T* at ca. 2.98 cm^3^ K mol^–1^ (**C**). Subsequently, the sample was heated at 0.3 K min^–1^ in the dark, then χ_M_*T* first increased, passing over a maximum at ca. 3.31 cm^3^ K mol^–1^, then it decreased practically to 0 cm^3^ K mol^–1^ at ca. 100 K (**D**) in two well-defined steps with characteristic *T*_LIESST_/*T*_TIESST_ values of 54.6 and 85.3 K. Upon heating up to 300 K (**E**), the sample recovers the HS state, and finally, cooling down to 10 K, the sample remains in the LS state (**F**). The hysteretic SCO behavior of **4** in these experimental conditions was centered *T*_1/2_ = 195.3 K, featuring a Δ*T*_hyst_ of 5.2 K ca. 60% narrower than that at 1 K min^–1^.

### Crystal Structures

**Figure 4:**
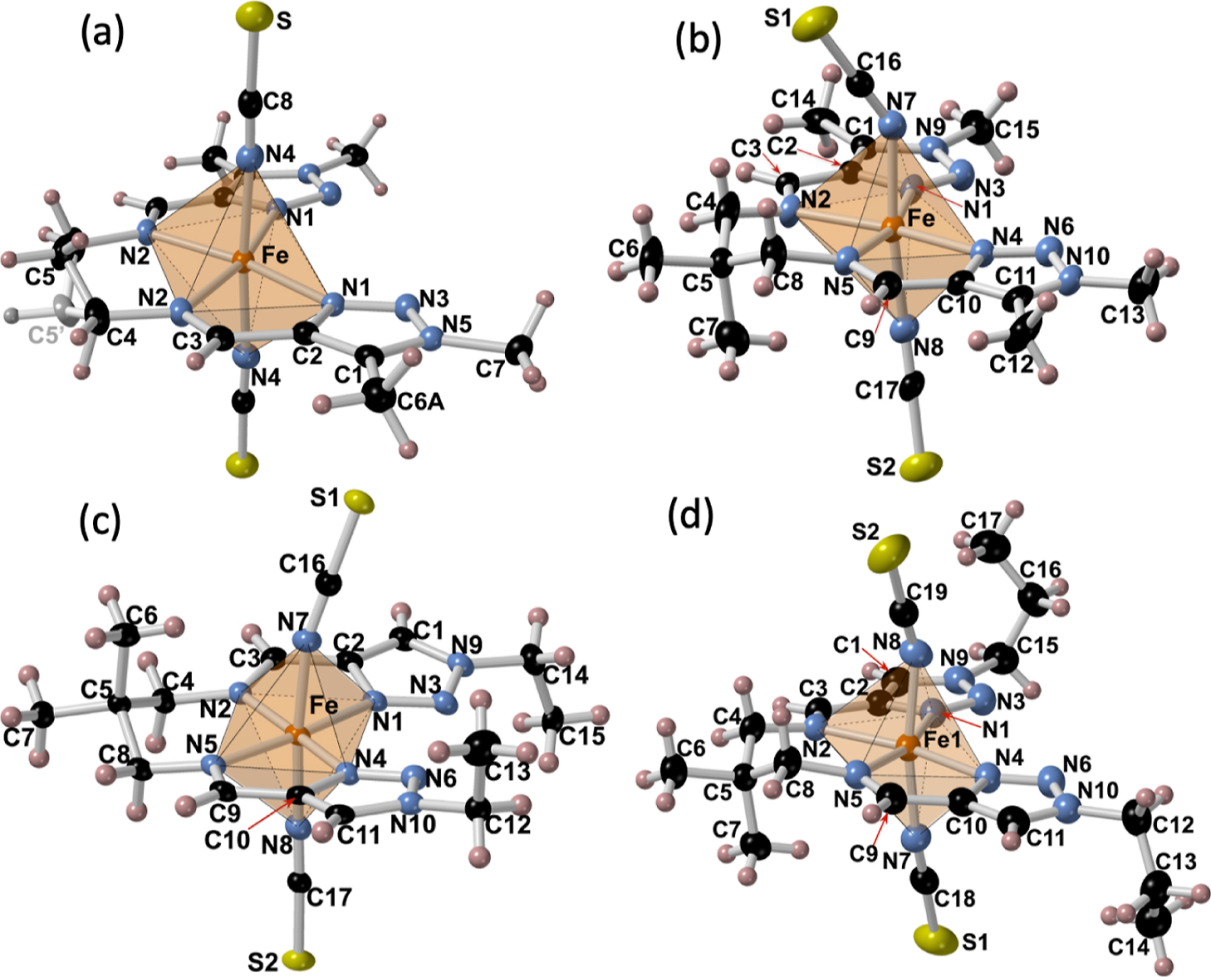
Molecular structure of **1** (250 K) (a), **2** (250 K) (b), **3** (120 K) (c), and **4** (220 K) (d). Thermal ellipsoids are represented at 30% probability.

The crystal structures of **1**–**4** have been analyzed in the LS and HS state. Compound **1** shows the orthorhombic *Fdd*2 space group in both spin states (LS 120 K and HS 250 K), while the other members of the series experience reversible symmetry breaking from orthorhombic *Fdd*2 (190 K) to monoclinic *Cc* (250 K), monoclinic *P*2_1_/*c* (120 K) to *I*2/*a* (280 K), and triclinic *P*1̅ (120 K) to monoclinic *P*2_1_/*c* (220 K) for **2**, **3,** and **4**, respectively, when switching from the LS to HS state. Furthermore, the HS state of compound **4** undergoes an additional phase transition just above 289.7 K (see Figure 2), adopting the triclinic space group *P*1̅ (300 K). Relevant crystal data for **1–4** are shown in Tables S1–S4. The corresponding bond lengths involving the [Fe^II^N_6_] octahedron together with the corresponding average ⟨Fe–N⟩ and the sum of the deviation from 90° of the 12 “cis” N–Fe–N angles, Σ = (|θ – 90|), are shown in Table 1 (the N–Fe–N angles are in Tables S5–S8). As usual, in this family of compounds, the coordination site of the Fe^II^ ion is defined by a distorted [FeN_6_] octahedron, with the tetradentate N_4_ ligand occupying the equatorial positions and the two SCN^–^ ligands completing the axial trans positions. Figure 4 displays the molecular structure for the four compounds together with the relevant atom numbering. The Fe–N bond lengths (Table 1) as well as the change of the average bond length [⟨Fe–N⟩ = 0.194 Å (**1**), 0.215 Å (**2**), 0.201 Å (**3**), and 0.207 or 0.200 Å (**4**)] upon SCO are perfectly consistent with the HS/LS states and the occurrence of a complete SCO, in agreement with the magnetic properties. The change of spin state is also reflected on the variation of the parameter Σ being ca. 19% (**1**), 38% (**2**), 30% (**3**), and 28% (**4**) larger in the HS state. In particular, the diagonal N–Fe–N bond angles of the equatorial plane significantly change upon SCO decreasing from ca. 174° (HS) down to ca. 164° (LS). In contrast, the axial N–Fe–N angle defined by the SCN^–^ groups marginally decreases by 0.69° from 175.86° (LS) when moving from the LS to the HS state in **1**. However, this decrease is more marked for **2** [4.89° from 178.87° (LS)], **3** [6.90° from 174.54° (LS)], and **4** [4.95° from 175.79° (LS)]. Furthermore, except for the φ[(S)C8–N4–Fe] tilt angle in **1** that modestly decreases by only Δφ = −2.1° from 175.4° (250 K, HS) to 173.3° (120 K, LS), in general, the Fe-NCS tilt angle (φ) is significantly affected by the SCO. Indeed, the changes are considerably much marked for **2** where φ[(S1)C16–N7–Fe] = 145.1° at 250 K (HS) increases up to 174.9° (190 K, LS) with Δφ = +29.8°, while Δφ is +5.3° for the opposite NCS^–^ group φ[(S2)C17–N8–Fe] = 169.6° (250 K, HS). As far as the complex **3** is concerned, the tilt angle defined by the two crystallographically distinct SCN^–^ groups in the LS state at 120 K (φ[(S1)C16–N7–Fe] = 161.1° and φ[(S2)C17–N8–Fe] = 177.8°) change in opposite direction with respect to that of the crystallographically unique SCN^–^ in the HS state at 280 K with φ[(S)C8–N4–Fe] ≈ 166.9° and Δφ = −5.8 and +10.9°, respectively. A similar situation is found for **4** where φ[(S1)C18–N7–Fe] = 165.4° (220 K, HS) and φ[(S2)C19–N8–Fe] = 152.7° (220 K, HS) which change, respectively, −4.0 and +21.1°, when the molecule adopts the LS state. For the high temperature HS phase, ϕ[(S1)C18–N7–Fe] = 167.2° (300 K, HS) and φ[(S2)C19–N8–Fe] = 164.1° (300 K, HS), which change, respectively, −1.8 and −11.4°, on going down to 220 K.

**Table 1:** Fe–N Bond Lengths, Average Bond Length, ⟨Fe–N⟩, and Angular Distortion (See Main Text) of the [FeN_6_] Octahedron

	**1**		**2**		**3**		**4**		
	120 K	250 K	190 K	250 K	120 K	280 K	120 K	220 K	300 K
Fe–N(1)	1.996(4)	2.220(5)	1.984(7)	2.219(8)	2.004(3)	2.247(4)	1.986(4)	2.234(2)	2.259(8)
Fe–N(2)	1.987(4)	2.166(4)	1.961(7)	2.173(8)	1.962(3)	2.167(5)	1.959(4)	2.189(2)	2.178(9)
Fe–N(4)	1.961(4)	2.140(5)	1.936(7)	2.227(8)	1.994(4)	2.078(6)	1.996(4)	2.254(2)	2.236(7)
Fe–N(5)				2.173(8)	1.961(3)		1.961(4)	2.174(2)	2.178(8)
Fe–N(7)				2.142(10)	1.954(4)		1.950(4)	2.081(2)	2.091(11)
Fe–N(8)				2.098(10)	1.938(4)		1.935(4)	2.101(2)	2.049(11)
**⟨Fe–N⟩**	1.981	2.175	1.960	2.172	1.968	2.164	1.965	2.172	2.165
Σ°	47.70	66.34	40.5	78.6	58.7	88.9	58.0	85.9	83.9

The C5 atom belonging to the propane-1,3-diamine moiety in **1** is disordered in two positions above and below the average plane containing C4 and the equatorial [FeN_4_] plane (Figure 4a), and this disorder is independent of the spin state. In contrast, the 2,2-dimethylpropane moiety in **2** is ordered in the HS state (250 K) (Figure 4b) but displays static disorder when switches to the LS state (190 K) (see Figure S1). The opposite situation occurs for **3** where the static disorder of the same moiety occurs in the HS state (see Figure S1), while no disorder occurs for **4**. In the latter compound the torsion angle, α, defined by the propyl groups significantly change with the spin state and the crystallographic phase. In particular, for N10C12C13C14, α moves from 59.45° (HS, 300 K) to 67.35° (HS, 220 K) and 62.51° (LS, 120 K) and for N9C15C16C17 from 60.55° (HS, 300 K) to 56.73° (HS, 220 K) and to 56.16° (LS, 120 K).

Minimized overlays of molecules **1–4** visualizing geometric changes due to the SCO are shown in Figure S4.

**Figure 5:**
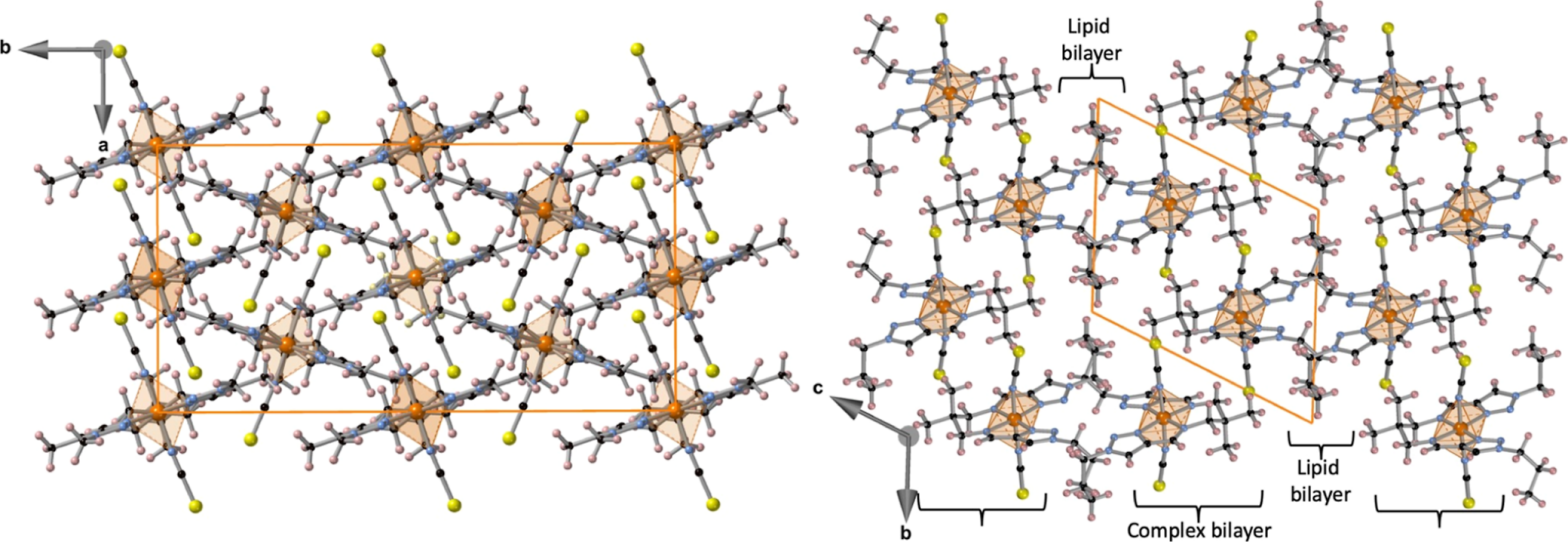
Crystal packing views of **2** (190 K, left) and **4** (120 K, right) down *c*- and *a*-axis, respectively.

The crystal packing of **1** and **2** is very similar, both in the HS and LS states, with the molecules tightly packed defining a herringbone pattern if viewed along axis *c* at 120 K (Figure 5 and Figure S2, left). Along *c* axis, the tilt angle defined by the equatorial plane of adjacent molecules separates from horizontality 14.78°/16.04° (**1**) and 17.35°/18.79° (**2**) in the HS/LS state. At 120 K, the shortest Fe···Fe intermolecular distances 7.622 and 7.784 Å for the complexes define a diamond-like lattice (Figure S3), which expand ca. 0.23 Å at 250 K in the HS state. However, the presence of the ethyl or propyl groups in **3** and **4**, respectively, attached to the triazole moieties modifies the crystal packing with respect to **1** and **2** (see Figures 5 and S2, right). Indeed, the molecules are organized in such a way that the pendant aliphatic chains define parallel lipid bilayer-like arrangements confining between them the more polar coordination core of the complexes, which also organize as double layers, in both cases lying parallel to the *a*–*b* plane. A common particularity is that each molecule is involved in the formation of an intricate 3D network of short contacts involving X···H (X = S, C and N) as well as C···C, S···C, and N···C pairs close and/or smaller than the sum of the Van der Waals radii which, as expected, most of them become shorter and larger in number when moving from the HS to the LS state (see Figures S5–S8). An alternative description and analysis of the packing of **1**, **2,** and **4** based on the energy framework methodology is given in the next section.

### Energy Framework Analysis

Understanding the relationship between the crystal structure and the physical properties of SCO complexes is a critical step in designing materials with desirable characteristics. One approach to studying crystal structures is through energy framework analysis, which is a new and convenient tool for evaluating the energetics of crystal structures and the SCO properties that arise from these structures. Energy framework analysis is a graphical method for analyzing the interaction energy landscape of crystal structures.^44,45^ The method involves representing centroids of molecules in the crystal structure as nodes, with intermolecular interactions being represented by cylindric connections between nodes. The cylinder radius is proportional to the magnitude of the interaction energy, which takes into account electrostatic, polarization, dispersion, and exchange–repulsion interaction contributions calculated using quantum mechanical methods based on B3LYP/6-31G(d,p) molecular wavefunctions (for details, see the Supporting Information). Initially created as a method having direct utility in exploring the mechanical properties of crystals, the energy framework analysis found its application also in the SCO researches. By constructing the energy difference framework (EDF) of the HS and the LS structures, it is possible to visualize changes in the interactions with the nearest neighboring surrounding resulting from changes in the molecular shape due to the SCO and the expansion/contraction of the lattice considering the complete set of intermolecular interactions. The EDF enables identification of the molecule–molecule contacts that is the most affected on transformation of the molecule and provides insight into the pathways of the cooperativity. Reeves et al.^46^ reported a pioneering study on the family of SCO complexes [Fe(PM-L)_2_(NCS)_2_], for which a linear variation of the transition abruptness with the sum of the magnitudes of the interaction energy changes within the first molecular coordination sphere in the crystal structure was found (PM-L denotes 4-substituted derivatives of the *N*-(2-pyridylmethylene)-4-aminobiphenyl ligand). Building on this seminal work, we aimed to apply the EDF analysis method to investigate the different hysteretic behavior observed in the title compounds, as well as in related complexes reported previously, which vary in the type of triazole group substituents.

**Figure 6:**
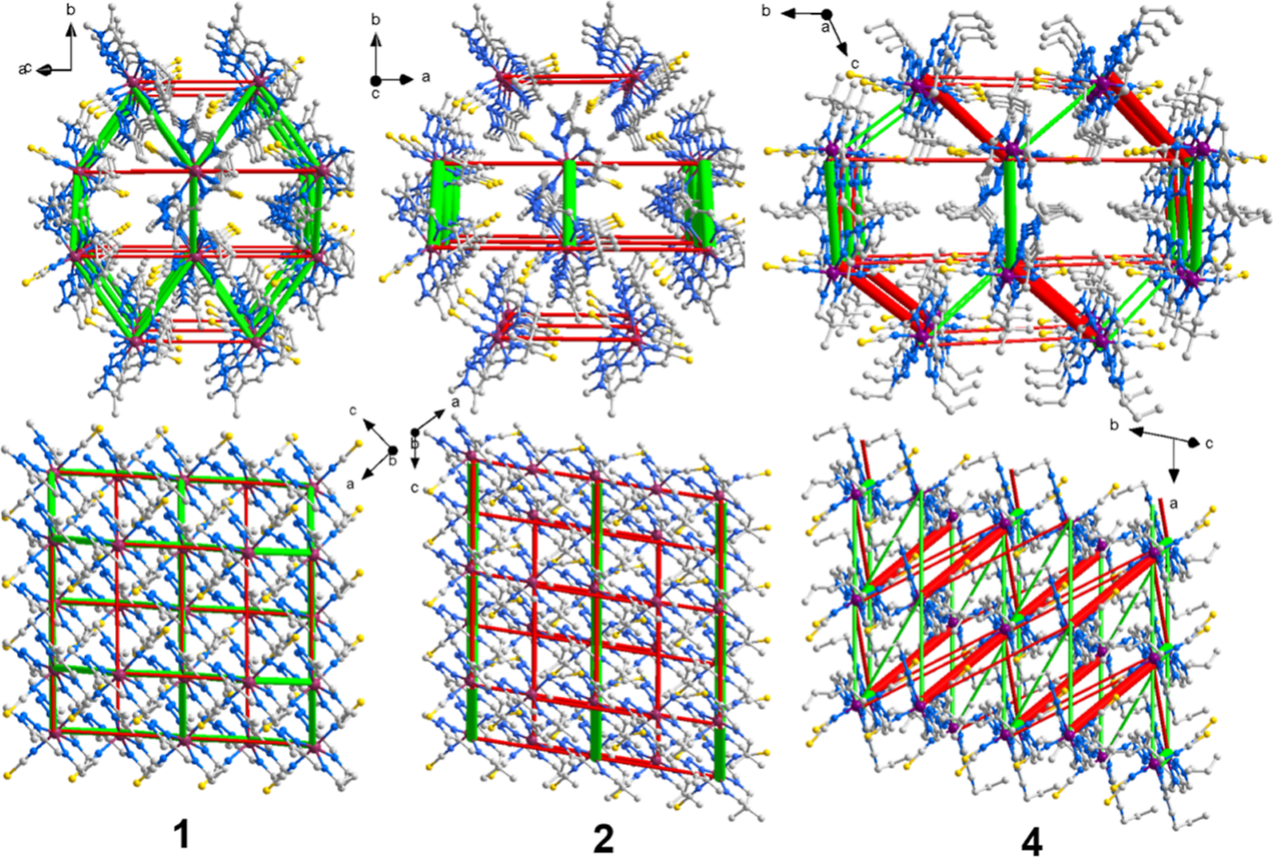
EDFs of the title compounds with one-step SCO, constructed using the values from the Tables S9–S11, column “Δ*E*(total)(HS–LS)”, and superimposed on a fragment of the HS phase crystal lattice viewed along the hexagonal channels (top) and perpendicular to their direction (bottom). The red cylinders correspond to weakening interactions and the green cylinders to the strengthening interactions. Tube size is scaled proportionally to the absolute value of the interaction energy; the cut-off is 5 kJ mol^–1^.

The present study of EDF includes the analysis of complexes **1**, **2**, and **4** that exhibit one-step SCO and have been characterized in both spin states. Although the complexes have similar molecular structures and exhibit similar crystal packing features (a hexagonal arrangement of molecules, see Figures 6, S9–S11), the lattice systematically expands as the number or size of the alkyl substituents increases. This expansion changes the interaction pattern between the nearest neighbor molecules that makes the topology and character of the EDFs different for **1**, **2,** and **4** (Figure 6).

If for **1** the EDF features a three-dimensional character, for **2** the lattice expansion due to the two methyl groups of the capping diamine results in a topology of EDF consisting of separate two-dimensional stacking layers (Figure 6). The amplitude of the molecule–molecule interaction change becomes more pronounced for **2** (see Figure 6). For **1,** the maximum positive and negative changes across the transition HS-to-LS lattice are −12.9 and +6.4 kJ mol^–1^, respectively, while for **2,** the values are −23.4 and +10.0 kJ mol^–1^, respectively. Namely, the presence of significant stabilizing and destabilizing lattice energy changes is attributed to more cooperative SCO transitions, while smaller changes are associated with less cooperative SCO transitions, as it was demonstrated for thiocyante complexes.^46^ Indeed, the hysteresis width is smaller for **1** (6 K) in comparison with **2** (13.6 K). For **4**, having the hysteresis of a similar width (13.5 K), the amplitude of the changes embraces values in the range −20.8 up to +25.1 kJ mol^–1^. For more details, see the Supporting Information, Tables S9–S11.

For comparison, we also constructed the EDFs of literature thiocyanate complexes based on 1-methyl-1,2,3-triazole-4-carbaldehyde with propane-1,3-diamine (**MeA** and **MeB**, which are polymorphs A and B, respectively),^37^ phenyl-1*H*-1,2,3-triazole-4-carbaldehyde (**Ph**), and *p*-toluyl-1*H*-1,2,3-triazole-4-carbaldehyde (**Tol**) with 2,2-dimethylpropane-1,3-diamine^38^ (see the Supporting Information, Tables S12–S15 and Figures S12–S15). The complex **Ph** shows a hysteresis of 20 K wide and has the most prominent energy changes −15.3 and +23.7 kJ mol^–1^. Meanwhile, introducing a methyl substituent to the phenyl group in the complex **Tol** results in a hysteresis 1 K wide and the energy change amplitude decreases down to −11.3 and +4.2 kJ mol^–1^. The two polymorphs, **MeA** and **MeB**, having identical hysteresis loops 2 K wide, slightly differ in terms of the range of interaction energy: the former shows an energy variations between −16.4 and +7.5 kJ mol^–1^, while the later between −14.8 and +8.5 kJ mol^–1^.

**Figure 7:**
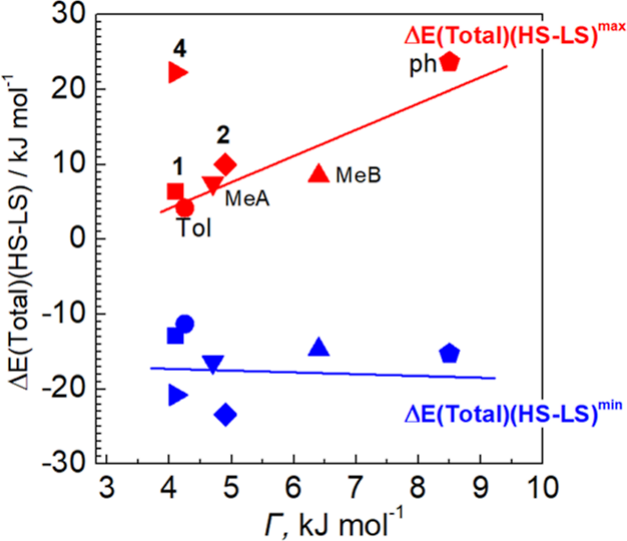
Extreme values of the EDF energies plot as a function of the cooperativity parameter Γ.

To find a correlation between the hysteretic behavior and the amplitude of the intermolecular energy change from the EDFs, we plotted the derived energy difference extrema against the cooperativity parameter Γ, calculated by fitting the hysteresis loops with the Slichter–Drickamer model with enthalpy and entropy fixed at experimental values (Figures 7 and S16). The obtained dependence clearly shows a systematic increasing trend for Δ*E*(total)(HS–LS)^max^ with increasing cooperativity, i.e., the higher the cooperativity, the larger the energy difference extrema, which is consistent with the conclusions of another team.^46^ Interestingly, the increase in energy variation during the SCO event between the closely related complexes **1** and **2** is achieved by introducing methyl groups in the capping diamine and thus changing the dimensionality of the EDF by activating/deactivating interaction pathways in the lattice (see Figure 6), which effectively leads to an increase in cooperativity in **2**. On the other hand, increasing the length of aliphatic substituent of the triazole moiety in **4** leads to an even larger value of Δ*E*(total)(HS–LS)^max^, while keeping the moderate cooperativity of the SCO transition. We tentatively attribute this deviation to a localization of the substantial structural changes on the terminal parts of the flexible propyl chains of the ligand (see Figure S4) on opposite, for example, to complex **Ph**, which exhibits similar energy difference values, but has rigid phenyl substituents and, presumably, better transmits large transformation of the lattice and of the surrounding of molecule to the SCO center.

The resulting correlation of lattice energy change and cooperativity is rather encouraging and even expected, but we emphasize that the chosen approach is oversimplified as the Slichter–Drickamer model does not distinguish between SCO coupled/uncoupled with symmetry breaking or order–disorder transitions, which, for example, are accounted in more sophisticated models.^47−49^ Finding the correlation between the EDF values and the precisely calculated cooperativity energy could be a topic for a future theoretical research.

## Discussion and Conclusions

Following the steps of Hagiwara et al., we have extended the family of SCO complexes [Fe(L)(*trans*-NCS)_2_] based on the tetradentate L ligand containing the 1,2,3-triazole donor moiety. In the first contribution, these authors reported an interesting example of polymorphism for the complex with L bearing R^1^ = Me, R^2^ = R^3^ = R^4^ = H, **MeA** and **MeB**, which radically differ in their crystal packing and *T*_1/2_, which is centered at 270 K (Δ*T*_hyst_ = 2 K) for **MeA** and 371 K (Δ*T*_hyst_ = 2 K) for **MeB**. The authors ascribed this large difference in *T*_1/2_ to the larger number of intermolecular contacts in **MeB**. It is worth noting that the unit cell volume and the corresponding variation of volume per Fe^II^ center of **MeB** are significantly larger than that of **MeA** (both with *Z* = 4). The crystal packing of polymorph **MeA** is very similar to that of the title compounds **1** [R^1^ = R^2^ = methyl, R^3^ = H, R^4^ = H] and **2** [R^1^ = R^2^ = R^3^ = methyl, R^4^ = H] (see Schemes 1d and 2), the major difference being the relative tilt angle defined by the equatorial coordination plane of adjacent complexes which is virtually zero for **MeA**. However, the *T*_1/2_ value for the latter is ca. 50 K higher than that of **1** and **2**, a fact that has not obvious justification, only, on the basis of molecular parameters such as ⟨Fe–N⟩ and Σ. For **MeA**, **1,** and **2,** the SCO is very abrupt, but the hysteresis loop, Δ*T*_hyst_ = 2 K, of the former is significantly smaller than that of **1** (Δ*T*_hyst_ = 6 K), reflecting the occurrence of shorter contacts in the latter. The difference in hysteresis width is more marked for **2** (Δ*T*_hyst_ = 13.6 K) due, in addition, to the larger number of short contacts involved. Furthermore, the SCO in **2** is synchronized with a crystallographic phase transition, which in turn is accompanied by order (HS)–disorder(LS) in the 2,2-dimethylpropane-1,3-diamine moiety. The order–disorder transition occurs in the opposite direction for **3** since the same moiety is ordered in the LS state and gets disordered in two positions in the HS state, a fact that is also accompanied by a synchronic crystallographic phase transition. In addition, an unusual reversible SCO is observed for **3** since during the HS → LS transformation, the first ca. 40% conversion is very steep and displays a relatively wide hysteresis, ca. 14 K wide, when completing the heating branch; however, when approaching 50% conversion, the slope decreases considerably, defining an inclined plateau and the second step characterized by ca. 5 K wide hysteresis. A plausible explanation for this behavior is that the structural changes favor the development of elastic frustration stemming from antagonistic attractive–repulsive intermolecular short contacts, thereby switching the nature of the SCO. It is interesting to remark that this “two-regime” SCO is also reflected in the relaxation of the photo-generated HS* state.

The 2,2-dimethylpropane-1,3-diamine moiety in **4** does not show significant changes at the temperatures investigated; however, the crystal displays a double crystallographic phase transition, namely, above 300 K, the crystal is the triclinic *P*1̅ space group and changes just below 300 K to the HS monoclinic *P*2_1_/*c* space group, and a reentrant transition to the triclinic phase takes place when changes to the LS phase below ca. 180 K. At variance with **3,** no frustration occurs and the SCO is comparable to that of **2**. The high-temperature phase transition is decoupled from the SCO but has important consequences on the TIESST and LIESST effects. The former generates a metastable phase defined by a 1:1 mixture of LS^q^ and HS^q^ monomers on quenching down to 10 K. The remaining LS^q^ molecules are transformed, via the subsequent LIESST effect, into a complete HS* phase. Then the resulting complete populated HS state corresponds to a 50% mixture of two distinct metastable HS^m^ = HS^q^ + HS* states. This fact is reflected on the relaxation of HS^m^, which is characterized by a two-step relaxation, each step reflecting different structural pathways, possibly differing in the positions of the propyl arms, as previously observed in related examples^50,51^ and probably in the φ(Fe–N–CS) angles. It is worth noting that the monoclinic HS state (220 K) affords a fully populated triclinic LS state, which seems to be the same as the 50% LS^q^ state obtained from the quenched triclinic HS phase via the TIESST effect since the corresponding the *T*_LIESST_ temperatures are similar.

The energy framework analysis, or its derivative energy difference framework analysis, presents an alternative approach to characterizing lattice interactions, as it considers all types of interactions and quantifies their strength in energy units across the SCO transition. This differs from the traditional approach, which solely focuses on the strongest interactions. From the analysis of the title complexes with single step transition (**1**, **2,** and **4**) and those reported by Hagiwara et al. (**MeA**, **MeB**, **Ph,** and **Tol**), it can be concluded that the expansion of the lattice due to the increasing volume of the substituents around the same SCO core can change the interaction patterns in a way that favors larger energy change on transformation between the spin states that is reflected on SCO behavior. By examining the highest and lowest values of energy difference in lattice interactions, it is possible to establish a satisfactory correlation with the cooperativity parameter Γ. In the case of title and literature complexes, the variation in energy is achieved through the breaking of symmetry and/or due to movable methyl groups in capping diamine group (**2** and **4**) or through the rotation of phenyl groups (**Ph**), which is made possible by a sufficiently flexible lattice that allows for occurrence of considerable structural transformations. Based on this, one can expect that more cooperative systems might be obtained for this type of complexes when using the 2,2-dimetylpropane moiety and N-pending groups that offer moderate strength bonding that can be broken or rearranged, for example, small rigid groups prone to hydrogen bonding, such as −COOH, −CONH_2_, five-membered heterocycles with N and NH groups, and so forth. Our laboratories are currently conducting further studies on *trans*-thiocyanate 1,2,3-triazole-based cooperative systems, and the results will be reported in due course.

In summary, we have presented a series of solvent-free *trans*-thiocyanate N-substituted 1,2,3-triazole-based complexes that exhibit SCO. Our results show that the hysteresis loop width of the studied complexes increases with the number or size of the aliphatic substituents. The differences between the behaviors of the complexes are explained in terms of the transition with or without symmetry breaking in the capping groups and changes in the intermolecular contacts. In addition, an energy analysis was performed to estimate the molecule–molecule energy in the lattice. The observed increase in cooperativity in the series and similar literature complexes were rationalized in terms of the amplitude of the energy change during the SCO transition.

## Experimental Section

### Materials

All chemicals were purchased from commercial suppliers and used without further purification (Merck, Enamine Ltd.). Complex [Fe(py)_4_(NCS)_2_] was synthesized following the method reported in the literature.^52^

### Synthesis of Complexes

Compounds **1–4** were obtained via ligand template procedure according to the following general scheme:
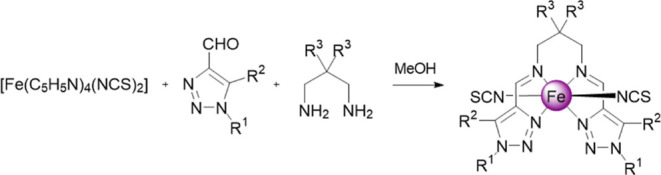


#### Synthesis of **1** (R^1^ = R^2^ = Me, R^3^ = H; L = *N*^1^,*N*^3^-Bis((1,5-dimethyl-1*H*-1,2,3-triazol-4-yl)methylene)propane-1,3-diamine)

To a boiling solution of [Fe(py)_4_(NCS)_2_] (100 mg, 0.2 mmol) and 1,5-dimethyl-1*H*-1,2,3-triazole-4-carbaldehyde (62 mg, 0.4 mmol) in MeOH (10 mL), 1,3-diaminopropane (17 μL, 0.2 mmol) was added. The orange solution formed was quickly filtered while hot through a plug of cotton and left overnight at room temperature. The orange crystals formed were filtered off and air dried. Yield 55 mg, 60%. Elemental analysis calcd (%) for C_15_H_20_FeN_10_S_2_: C, 39.13; H, 4.38; N, 30.43. Found: C, 39.21; H, 4.17; N, 30.22.

#### Synthesis of **2** (R^1^ = R^2^ = Me, R^3^ = Me; L = *N*^1^,*N*^3^-Bis((1,5-dimethyl-1*H*-1,2,3-triazol-4-yl)methylene)-2,2-dimethylpropane-1,3-diamine)

To a boiling solution of [Fe(py)_4_(NCS)_2_] (100 mg, 0.2 mmol) and 1,5-dimethyl-1*H*-1,2,3-triazole-4-carbaldehyde (62 mg, 0.4 mmol) in MeOH (10 mL), 2,2-dimethylpropane-1,3-diamine (24 μL, 0.2 mmol) was added. The orange-brown solution formed was quickly filtered while hot through a plug of cotton and left overnight at room temperature. The orange crystals formed were filtered off and air dried. Yield 62 mg, 64%. Elemental analysis calcd (%) for C_17_H_24_FeN_10_S_2_: C, 41.81; H, 4.95; N, 28.68. Found: C, 41.98; H, 4.74; N, 28.60.

#### Synthesis of **3** (R^1^ = C_2_H_5_, R^2^ = H, R^3^ = Me; L = *N*^1^,*N*^3^-Bis((1-ethyl-1*H*-1,2,3-triazol-4-yl)methylene)-2,2-dimethylpropane-1,3-diamine)

To a boiling solution of [Fe(py)_4_(NCS)_2_] (100 mg, 0.2 mmol) and 1-ethyl-1*H*-1,2,3-triazole-4-carbaldehyde (62 mg, 0.4 mmol) in MeOH (10 mL), 2,2-dimethylpropane-1,3-diamine (24 μL, 0.2 mmol) was added. The orange-brown solution formed was quickly filtered while hot through a plug of cotton and left overnight at room temperature. The orange crystals formed were filtered off and air dried. Yield 61 mg, 63%. Elemental analysis calcd (%) for C_17_H_24_FeN_10_S_2_: C, 41.81; H, 4.95; N, 28.68. Found: C, 41.88; H, 4.85; N, 28.56.

#### Synthesis of **4** (R^1^ = C_3_H_7_, R^2^ = H, R^3^ = Me; L = *N*^1^,*N*^3^-Bis((1-propyl-1*H*-1,2,3-triazol-4-yl)methylene)-2,2-dimethylpropane-1,3-diamine)

To a boiling solution of [Fe(py)_4_(NCS)_2_] (100 mg, 0.2 mmol) and 1-propyl-1*H*-1,2,3-triazole-4-carbaldehyde (71 mg, 0.4 mmol) in MeOH (10 mL), 2,2-dimethylpropane-1,3-diamine (24 μL, 0.2 mmol) was added. The orange-brown solution formed was quickly filtered while hot through a plug of cotton and left overnight at room temperature. The orange crystals formed were filtered off and air dried. Yield 56 mg, 54%. Elemental analysis calcd (%) for C_19_H_28_FeN_10_S_2_: C, 44.19; H, 5.46; N, 27.12. Found: C, 44.22; H, 5.35; N, 27.24.

### Physical Characterization

Variable-temperature magnetic susceptibility data (15–20 mg) were recorded on samples at variable rates between 10 and 400 K using a Quantum Design MPMS2 SQUID susceptometer operating at 1 T magnet. The LIESST experiments were performed at 10 K in a commercial sample holder (Quantum Design Fiber Optic Sample Holder), wherein a quartz bucket containing ca. 1 mg of a sample was held against the end of a quartz fiber coupled with a red laser (633 nm, 15 mW cm^–1^). After reaching the saturation of susceptibility, the sample was heated up at the rate 0.3 K min^–1^. The raw data were corrected for a diamagnetic background arising from the sample holder. The resulting magnetic signal was calibrated by scaling to match values with those of bulk sample. DSC measurements were performed on a Mettler Toledo TGA/SDTA 821e under a nitrogen atmosphere with a rate of 10 K min^–1^. The raw data were analyzed with the Netzsch Proteus software with an overall accuracy of 0.2 K in the temperature and 2% in the heat flow. Elemental CHN analysis was performed after combustion at 850 °C using IR detection and gravimetry by means of a PerkinElmer 2400 series II device. Single crystal X-ray diffraction data of **1–4** were collected on a Nonius Kappa-CCD single crystal diffractometer using graphite mono-chromated Mo Kα radiation (λ = 0.71073 Å). A multi-scan absorption correction was performed. The structures were solved by direct methods using SHELXS-2014 and refined by full-matrix least squares on *F*^2^ using SHELXL-2014.^53^ Non-hydrogen atoms were refined anisotropically, and hydrogen atoms were placed in calculated positions refined using idealized geometries (riding model) and assigned fixed isotropic displacement parameters. CCDC files, 2248042–2248050, contain the supplementary crystallographic data for this paper. These data can be obtained free of charge from The Cambridge Crystallographic Data Centre via www.ccdc.cam.ac.uk/data_request/cif. Energy framework analysis calculation were performed by using *CrystalExplorer21*.^45^ Electrostatic potential and intermolecular interaction energies, which were partitioned into electrostatic, polarization, dispersion, and repulsion energy components, were calculated based on the B3LYP/6-31G(d,p) wave functions that were obtained by using the structural data from the corresponding CIF files. The obtained interaction energies were further utilized to map the network of energy frameworks across different pairs. The radii of these cylinders are directly proportional to the strength of the corresponding intermolecular interactions.

## Supporting Information Available

Details of the crystal data collection and refinement parameters as well as selected bond distances and angles, full color-coded interaction mappings of a central reference molecule with the nearest neighbors and the contributions to the total energy in both spin states for **1**–**4,** molecular structure of **2** and **3** at 190 and 280 K, respectively, crystal packing of **1** and **3** at 120 K and **2** at 190 K, overlay of molecules **1–4** in the LS and HS states, intermolecular contacts for **1**–**4,** and energy frameworks (PDF)
