# Supplementary material for: Order–Disorder, Symmetry Breaking, and Crystallographic Phase Transition in a Series of Bis(trans-thiocyanate)iron(II) Spin Crossover Complexes Based on Tetradentate Ligands Containing 1,2,3-Triazoles
Source: Inorg Chem. 2023 May 25;62(23):9044–53. doi: 10.1021/acs.inorgchem.3c00830 (PMC10265696; doi:10.1021/acs.inorgchem.3c00830)
Supplement: Supplementary file 1 — ic3c00830_si_001.pdf [file ic3c00830_si_001.pdf]

Supporting Information

Order-disorder, symmetry breaking and crystallographic phase transition in a series of bis(trans-thiocyanate)iron(II) spin crossover complexes based on tetradentate ligands containing 1,2,3-triazoles

Maksym Seredyuk,[a,b] Kateryna Znovjyak,[b] Francisco Javier Valverde-Muñoz,[a] M. Carmen Muñoz,[c] Volodymyr M. Amirkhanov,[b] Igor O. Fritsky,[b] Jose A. Real[a]

[a]*Departament de Quimica Inorgànica, Institut de Ciencia Molecular (ICMol), Universidad de Valencia, Valencia, Spain. E-mail: jose.a.real@uv.es*
[b]*Department of Chemistry, Taras Shevchenko National University of Kyiv, 64/13, Volodymyrska Street, 01601 Kyiv, Ukraine. E-mail: maksym.seredyuk@uv.es, maksym.seredyuk@knu.ua*
[c]*Departamento de Fisica Aplicada, Universitat Politècnica de València, Camino de Vera s/n, 46022 Valencia, Spain*

Contents

Crystallographic data of **1**-**4** (**Tables S1**-**S4**) **S2-5**

Selected bond lengths [Å] and angles [°] of **1**-**4** (**Tables S5**-**S8**) **S6-7**

Molecular structure of **2** (190 K) and **3** (280 K) (**Figure S1**) and crystal packing of **1** and **3** (**Figure S2**) **S8**

Crystal packing illustrating the diamond-like arrangement characteristic of complexes **1** and **2** (**Figure S3**) **S9**

Minimized overlay of molecules **1**-**4**, visualizing structural changes due the SCO (**Figure S4**) **S9**

Intermolecular contacts for **1**-**4** in the LS and HS states (**Figures S5**-**S8**) **S10-13**

Energy frameworks and energy difference frameworks for **1**, **2**, **4**, **Ph**, **Tol**, **MeA** and **MeB** (**Tables S9**-**S15** and **Figures S9**-**S15**, respectively) **S14-27**

Slichter-Drickamer model fitting of magnetic data for **1, 2, 4, Ph, Tol, MeA** and **MeB** **S28**

References **S28**

Table S1. Crystal data of **1**.

	**120 K**	**250 K**
Empirical formula	$C_{15}H_{20}N_{10}S_2Fe$	
*M*r	460.38	454.33
Crystal system	orthorhombic	
Space group	*Fdd*2	
a (Å)	12.1965(4)	12.4278(5)
b (Å)	24.3885(10)	25.6147(12)
c (Å)	13.6364(4)	13.2533(5)
V (Å^3)	4056.2(2)	4219.0(3)
Z	8	
D_c (mg cm^{-3})	1.508	1.431
F(000)	1904	1856
μ (Mo-K$_\alpha$) (mm^{-1})	0.973	0.935
Crystal size (mm)	0.03x0.05x0.07	
No. of total reflections	2562	2723
No. of reflections [$I>2\sigma(I)$]	2119	1992
R [$I>2\sigma(I)$]	0.0466	0.0554
wR [$I>2\sigma(I)$]	0.1013	0.1161
S	1.012	1.073

$R_1 = \Sigma ||Fo| - |Fc|| / \Sigma |Fo|$; $wR = [\Sigma [w(Fo^2 - Fc^2)^2] / \Sigma [w(Fo^2)^2]]^{1/2}$;
$w = 1/[\sigma^2(Fo^2) + (m P)^2 + n P]$ where $P = (Fo^2 + 2Fc^2) / 3$;
m = 0.0511 (**1**) and 0.0588 (**2**);
n = 11.1661 (**1**) and 4.7268 (**2**)

Table S2. Crystal data of **2**.

	**190 K**	**250 K**
Empirical formula	$C_{17}H_{24}N_{10}S_2Fe$	
Mr	488.43	
Crystal system	orthorhombic	monoclinic
Space group	*Fdd*2	*Cc*
a (Å)	12.4604(9)	12.5718(14)
b (Å)	24.223(2)	25.595(2)
c (Å)	15.0789(11)	9.1235(9)
β(°)		126.911(7)
V (Å^3)	4551.3(6)	2347.3(4)
Z	8	4
D_c (mg cm^{-3})	1.426	1.382
F(000)	2032	1016
μ (Mo-K$_\alpha$) (mm^{-1})	0.872	0.845
Crystal size (mm)	0.03x0.04x0.08	
No. of total reflections	2105	4080
No. of reflections [$I>2\sigma(I)$]	1131	2159
R [$I>2\sigma(I)$]	0.0629	0.0599
wR [$I>2\sigma(I)$]	0.0742	0.1231
S	1.012	0.924

$R_1 = \Sigma\,||Fo| - |Fc||\,/\,\Sigma\,|Fo|$; $wR = [\,\Sigma\,[w(Fo^2 - Fc^2)^2]\,/\,\Sigma\,[w(Fo^2)^2]]^{1/2}$;
$w = 1/\,[\sigma^2(Fo^2) + (\mathrm{m}\,P)^2 + \mathrm{n}\,P]$ where $P = (Fo^2 + 2Fc^2)\,/\,3$;
m = 0.0128 (**1**) and 0.0749 (**2**);
n = 0.0000 (**1**) and 0.0000 (**2**)

Table S3. Crystal data of **3**.

	**120 K**	**280 K**
Empirical formula	$C_{17}H_{24}N_{10}S_2Fe$	
Mr	488.43	
Crystal system	monoclinic	
Space group	$P2_1/c$	$I2/a$
a (Å)	12.0799(4)	9.0781(6)
b (Å)	23.2294(8)	22.6564(14)
c (Å)	8.3091(3)	12.5000(7)
β(°)	105.418(4)	108.182(7)
V (Å^3)	2247.70(14)	2442.6(3)
Z	4	
D_c (mg cm^{-3})	1.443	1.328
F(000)	1016	
μ (Mo-K$_\alpha$) (mm^{-1})	0.883	0.812
Crystal size (mm)	0.04x0.08x0.10	
No. of total reflections	5684	2143
No. of reflections [$I>2\sigma(I)$]	3485	955
R [$I>2\sigma(I)$]	0.0587	0.0610
wR [$I>2\sigma(I)$]	0.1312	0.1019
S	0.869	1.026

$R_1 = \Sigma ||Fo| - |Fc|| / \Sigma |Fo|$; $wR = [\Sigma [w(Fo^2 - Fc^2)^2] / \Sigma [w(Fo^2)^2]]^{1/2}$.
$w = 1/ [\sigma^2(Fo^2) + (m\ P)^2 + n\ P]$ where $P = (Fo^2 + 2Fc^2) / 3$;
m = 0.0855 (**1**) and 0.0303 (**2**);
n = 5.3084 (**1**) and 0.6387 (**2**)

Table S4. Crystal data of **4**.

	**120 K**	**220 K**	**300 K**
Empirical formula		$C_{19}H_{28}N_{10}S_2Fe$	
*M*r		516.48	
Crystal system	triclinic	monoclinic	triclinic
Space group	*P*-1	$P2_1/c$	*P*-1
a (Å)	8.524(6)	8.917(3)	8.91(2)
b (Å)	12.024(8)	23.956(7)	12.63(3)
c (Å)	13.585(9)	12.475(4)	13.54(3)
α (°)	112.46(4)		109.99(7)
β(°)	96.29(3)	108.279(11)	98.72(7)
γ(°)	103.99(3)		107.73(7)
V ($Å^3$)	1216.5(15)	2530.4(14)	1308(5)
Z	2	4	2
D_c (mg cm^{-3})	1.410	1.356	1.311
F(000)	540	1080	540
μ (Mo-K_α) (mm^{-1})	0.820	0.788	0.762
Crystal size (mm)		0.06x0.10x0.10	
No. of total reflections	5532	5789	7447
No. of reflections [$I>2\sigma(I)$]	4366	4468	2236
R [$I>2\sigma(I)$]	0.0592	0.0368	0.1217
wR [$I>2\sigma(I)$]	0.1364	0.0919	0.3515
S	1.161	1.143	1.207

$R_1 = \Sigma ||Fo| - |Fc|| / \Sigma |Fo|$; $wR = [\Sigma [w(Fo^2 - Fc^2)^2] / \Sigma [w(Fo^2)^2]]^{1/2}$.
$w = 1/ [\sigma^2(Fo^2) + (m P)^2 + n P]$ where $P = (Fo^2 + 2Fc^2) / 3$;
m = 0.0069 (**1**), 0.0463 (**2**) and 0.2000 (**3**);
n = 6.4584 (**1**), 1.3760 (**2**) and 0.0000 (**3**)

Table S5. Selected bond lengths [Å] and angles [°] of **1**.

	**120 K**	**250 K**
N(1)-Fe-N(2)	80.8(2)	76.4(2)
N(1)-Fe-N(4)	88.0(2)	89.5(2)
N(2)-Fe-N(4)	90.8(2)	92.7(2)

Table S6. Selected bond lengths [Å] and angles [°] of **2**.

	**190 K**	**250 K**
N(1)-Fe-N(2)	81.3(2)	76.8(3)
N(1)-Fe-N(4)	89.5(3)	118.4(3)
N(1)-Fe-N(5)		165.0(3)
N(1)-Fe-N(7)		89.7(3)
N(1)-Fe-N(8)		88.4(3)
N(2)-Fe-N(4)		164.7(3)
N(2)-Fe-N(5)		88.2(3)
N(2)-Fe-N(7)		88.3(4)
N(2)-Fe-N(8)		96.9(4)
N(4)-Fe-N(5)		76.5(3)
N(4)-Fe-N(7)		90.4(3)
N(4)-Fe-N(8)		85.5(3)
N(5)-Fe-N(7)		88.5(3)
N(5)-Fe-N(8)		94.8(3)
N(7)-Fe-N(8)		174.0(4)

Table S7. Selected bond lengths [Å] and angles [°] of **3**.

	**120 K**	**280 K**
N(1)-Fe-N(2)	81.12(14)	75.8(2)
N(1)-Fe-N(4)	104.91(14)	87.8(2)
N(1)-Fe-N(5)	174.25(14)	
N(1)-Fe-N(7)	84.29(14)	
N(1)-Fe-N(8)	91.25(14)	
N(2)-Fe-N(4)	172.40(14)	97.0(2)
N(2)-Fe-N(5)	93.14(14)	
N(2)-Fe-N(7)	89.74(14)	
N(2)-Fe-N(8)	92.70(15)	
N(4)-Fe-N(5)	80.84(14)	
N(4)-Fe-N(7)	86.32(14)	
N(4)-Fe-N(8)	91.79(14)	
N(5)-Fe-N(7)	95.99(14)	
N(5)-Fe-N(8)	88.76(14)	
N(7)-Fe-N(8)	174.53(15)	

Table S8. Selected coordination bond angles [°] of **4**.

	**120 K**	**220 K**	**300 K**
N(1)-Fe-N(2)	81.1(2)	76.39(7)	76.1(3) 13.9
N(1)-Fe-N(4)	104.7(2)	119.61(7)	119.9(3) 29.9
N(1)-Fe-N(5)	174.4(2)	164.65(7)	164.4(3)
N(1)-Fe-N(7)	84.0(2)	84.96(8)	85.3(3) 4.7
N(1)-Fe-N(8)	92.8(2)	90.69(8)	89.2(3) 0.69
N(2)-Fe-N(4)	173.43(14)	163.97(7)	164.0(3)
N(2)-Fe-N(5)	93.4(2)	88.33(7)	88.6(4) 1.4
N(2)-Fe-N(7)	90.5(2)	94.50(8)	94.0(4) 4.0
N(2)-Fe-N(8)	91.7(2)	92.30(8)	91.5(4) 1.5
N(4)-Fe-N(5)	80.8(2)	75.70(7)	75.4(3) 14.6
N(4)-Fe-N(7)	87.1(2)	86.60(8)	88.7(3). 1.3
N(4)-Fe-N(8)	91.2(2)	88.56(8)	88.0(3) 2.0
N(5)-Fe-N(7)	95.3(2)	97.85(8)	98.5(4) 8.5
N(5)-Fe-N(8)	88.2(2)	88.46(8)	88.6(4) 1.4
N(7)-Fe-N(8)	175.8(2)	170.84(9)	171.1(3)

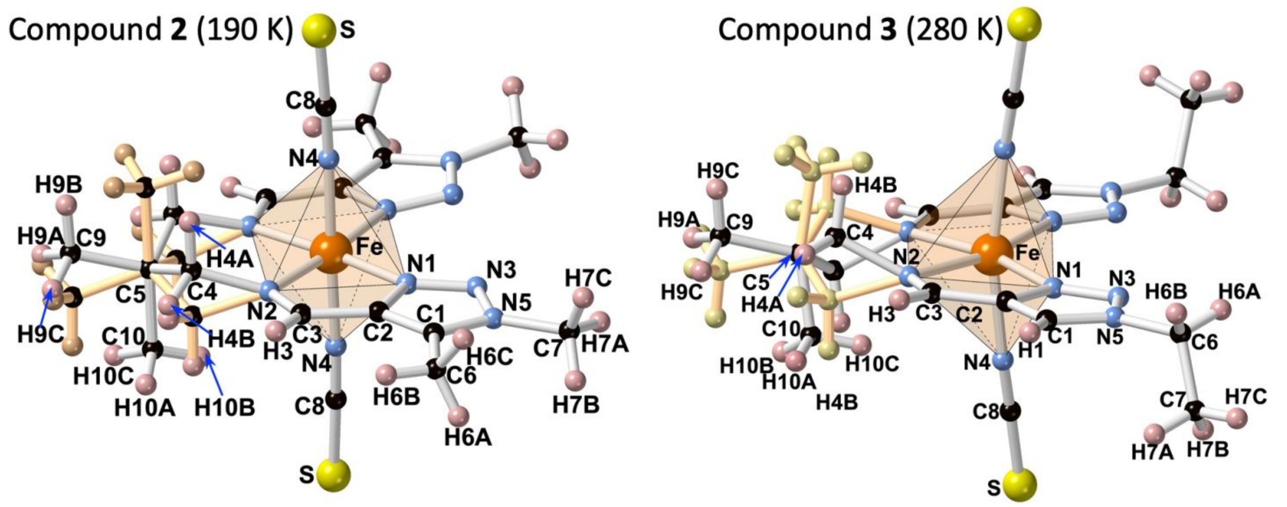

Figure S1. Molecular structure of **2** (190 K) (left) and **3** (280 K) (right) displaying the static disorder in the LS at 190 K. The two possible configurations are colored differently.

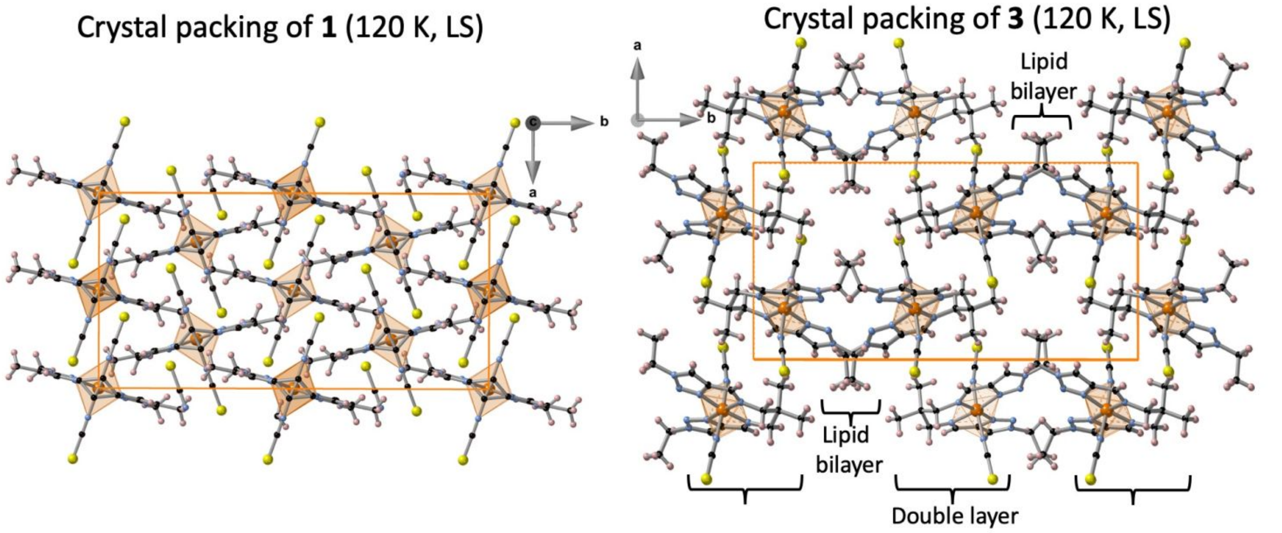

Figure S2. Crystal packing of **1** (left) and **3** (right) at 120 K in the LS state.

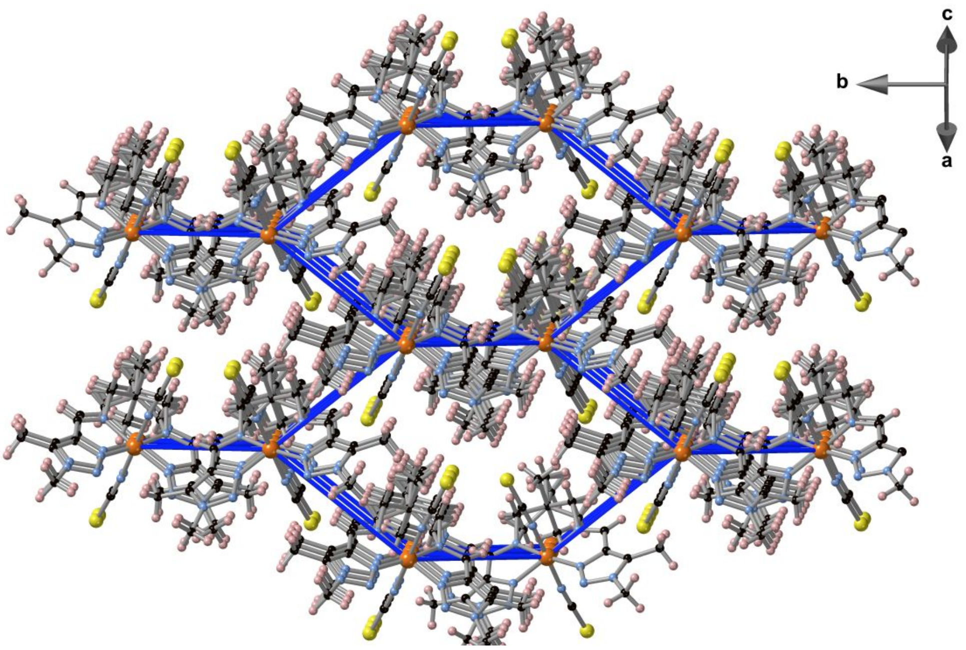

Figure S3. Crystal packing view of **2** (190 K) illustrating the diamond-like arrangement of the complexes. This packing is representative of the HS and LS states of **1** and **2**.

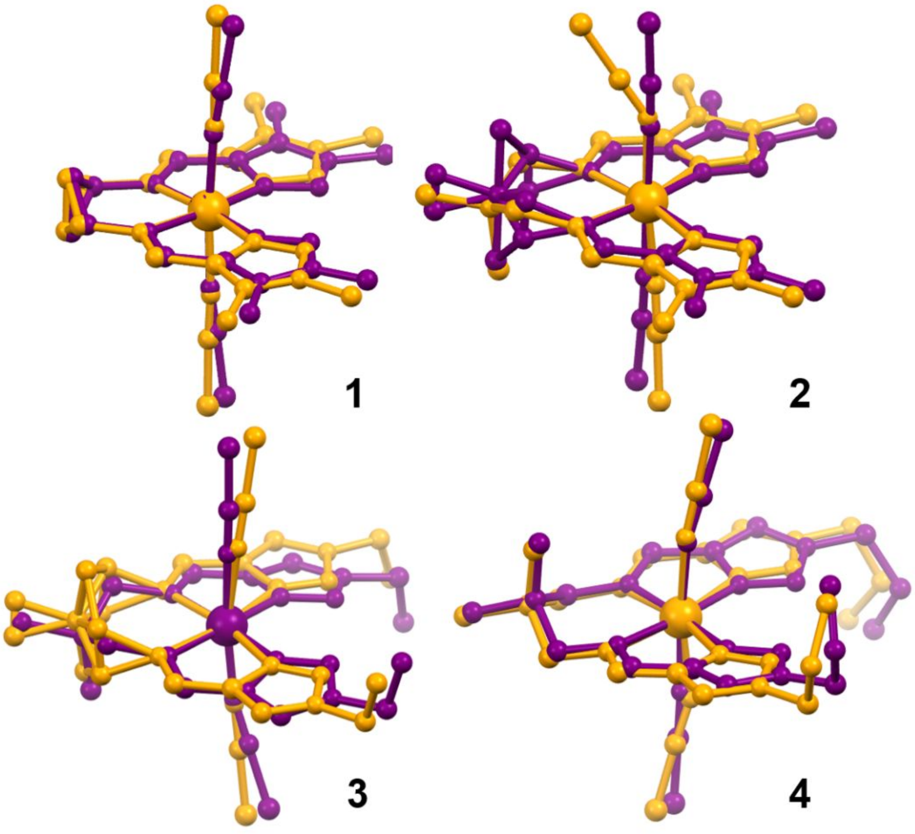

Figure S4. Minimized overlay of molecules **1**-**4**, visualizing structural changes due the SCO between the LS (purple) and HS (orange) spin states.

1 (120 K, LS)		
Intermolecular Contact	length	Length-VdW
H6C···C8	2.567	-0.333
H7C···S	2.698	-0.302
H3···C8	2.616	-0.284
C1···C3	3.505	+0.105
C1···C7	3.518	+0.118
C2···C7	3.485	+0.085
C3···C8	3.462	+0.062
C6···C8	3.451	+0.051
C3···S	3.548	+0.048
C6···S	3.681	+0.181
C7···S	3.646	+0.146

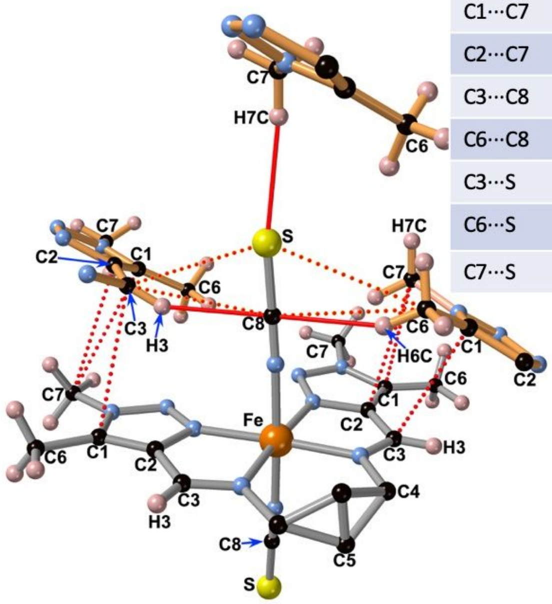

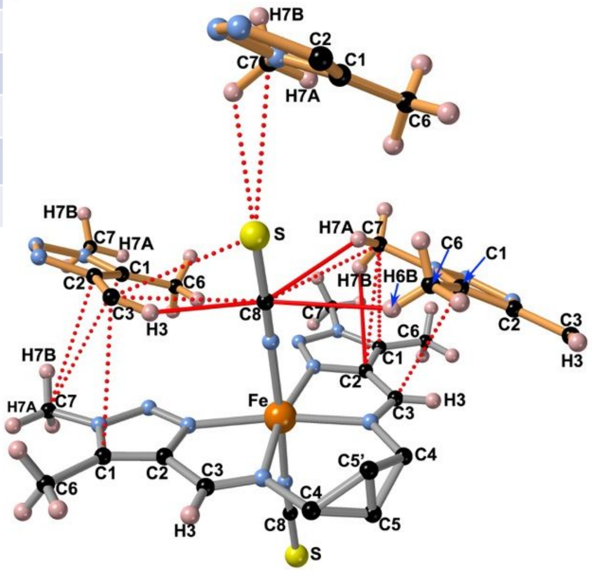

1 (250 K, HS)		
Intermolecular Contact	length	Length-VdW
H7A···C8	2.818	-0.082
H7B···C2	2.833	-0.067
H6B···C8	2.843	-0.057
H3···C8	2.617	-0.283
H7C···S	3.005	0.005
H3···S	3.094	0.094
H5B···C7	2.995	0.095
C1···C3	3.589	+0.189
C1···C7	3.807	+0.407
C2···C7	3.577	+0.177
C3···C8	3.462	+0.062
C6···C8	3.669	+0.269
C3···S	3.646	+0.146
C6···S	3.862	+0.362
C7···S	3.590	+0.090

Figure S5. Intermolecular contacts for **1** at 120 K (LS) (left) and 250 K (HS) (right). Solid and dotted lines represent, respectively, interatomic contacts shorter and slightly larger than the sum of VdW radii. Only the upper half of the contacts are shown for simplicity. Note that the column Length-VdW gives the difference between the actual interatomic length and the expected for the corresponding VdW radii.

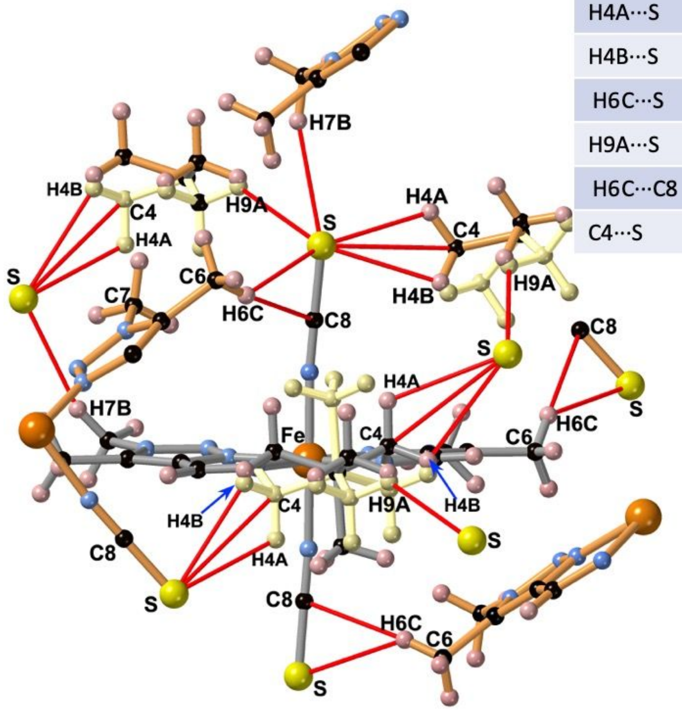

2 (190 K, LS)		
Intermolecular Contact	length	Length-VdW
H7B···S	2.770	-0.230
H4A···S	2.903	-0.0972
H4B···S	2.939	-0.061
H6C···S	2.909	-0.091
H9A···S	2.952	-0.048
H6C···C8	2.810	-0.090
C4···S	3.385	-0.115

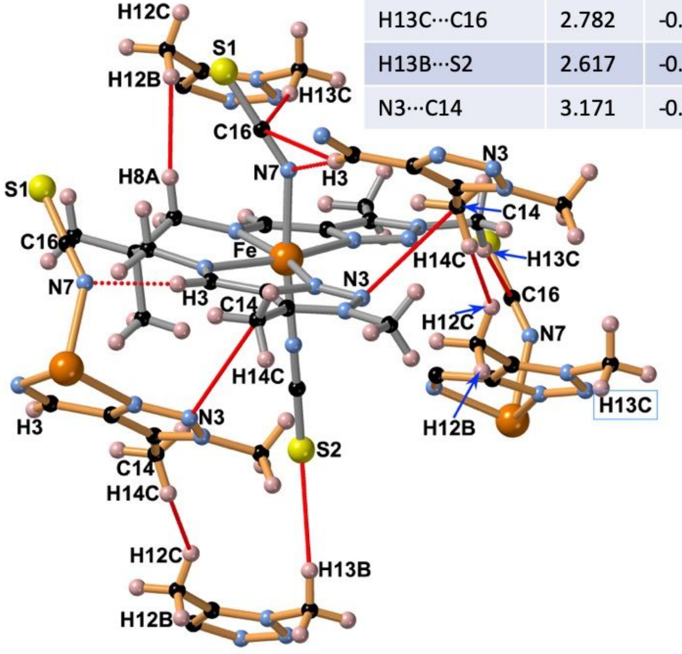

2 (250 K, HS)		
Intermolecular Contact	length	Length-VdW
H3···N7	2.262	-0.128
H3···C16	2.877	-0.023
H13C···C16	2.782	-0.118
H13B···S2	2.617	-0.283
N3···C14	3.171	-0.079

Figure S6. Intermolecular contacts for **2** (190 K, LS) (left) and 250 K (HS) (right). Solid and dotted lines represent, respectively, interatomic contacts shorter and slightly larger than the sum of VdW radii. Note that the column Length-VdW gives the difference between the actual interatomic length and the sum of the corresponding VdW radii.

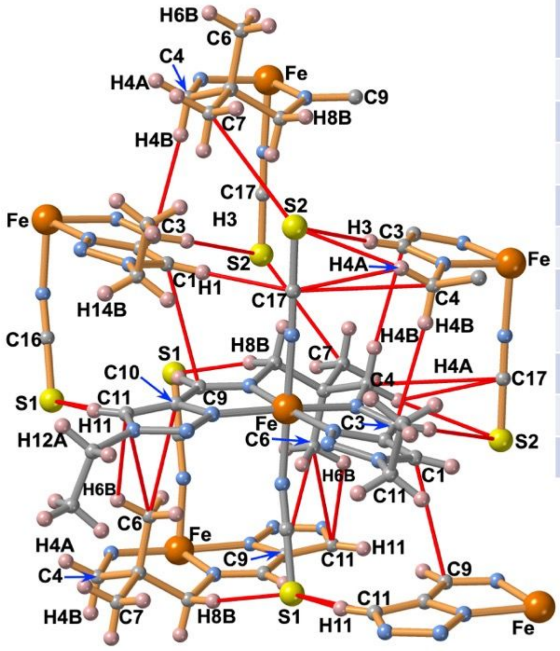

3 (120 K, LS)		
Intermolecular Contact	length	Length-VdW
H11···S1	2.783	-0.217
H1···C17	2.639	-0.261
H8B···S1	2.781	-0.219
H3···S2	2.893	-0.107
H4A···S2	2.841	-0.159
H4A···C17	2.758	-0.142
H12A···N6	2.535	-0.215
H14B···N6	2.444	-0.306
H6B···C11	2.887	-0.013
H4B···C3	2.834	-0.066

3 (280 K, HS)		
Intermolecular Contact	length	Length-VdW
H1···S	2.857	-0.143
H6B···N3	2.594	-0.156
H3···S	2.924	-0.076
H4'A···S	2.992	-0.008
H4A···S	2.982	-0.018
H4A···C8	2.875	-0.025
H10B···C1	2.840	-0.060
C2···C10	3.380	-0.020

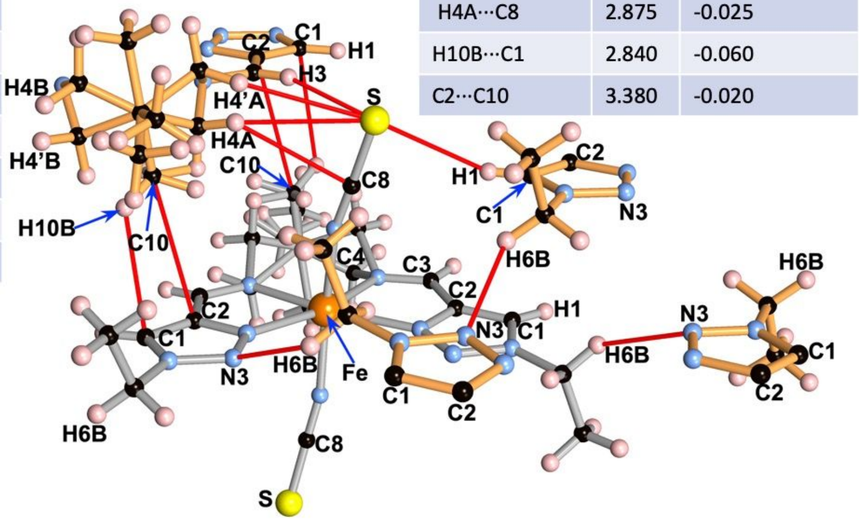

Figure S7. Intermolecular contacts for **3** (120 K, LS) (left) and 280 K (HS) (right). Solid lines represent interatomic contacts shorter than the sum of VdW radii. Note that the column Length-VdW gives the difference between the actual interatomic length and the sum of the corresponding VdW radii. Only the upper half of the contacts are shown for simplicity at 280 K (right).

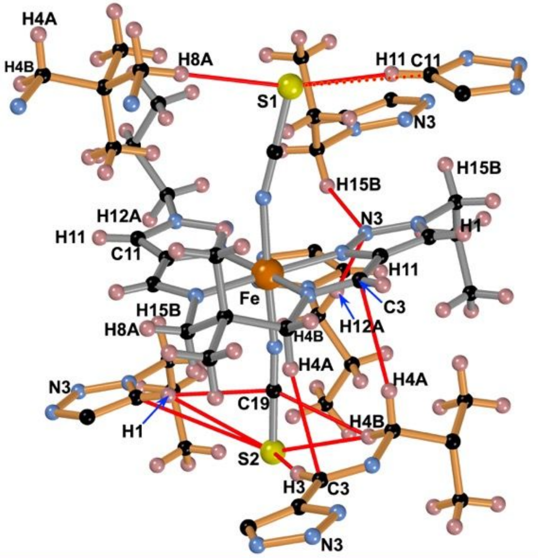

4 (120 K, LS)					
Contact	length	Length-VdW	Contact	length	Length-VdW
H1···S2	2.941	-0.059	H12A···N3	2.439	-0.311
H11···S1	2.790	-0.210	H15B···N3	2.668	-0.082
C19···H1	2.680	-0.220	S2···C1	3.685	0.185
H3···S2	2.896	-0.104	S1···C11	3.626	0.126
H4B···S2	2.932	-0.068			
H4A···C3	2.846	-0.054			
H4B···C19	2.802	-0.098			
H8A···S1	2.794	-0.206			

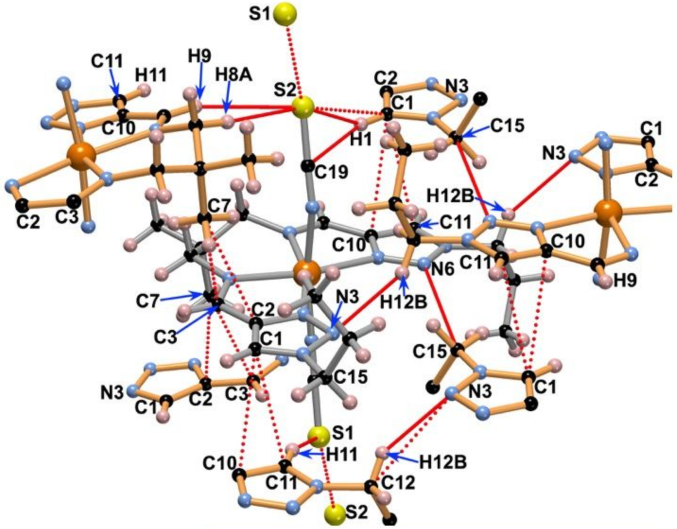

4 (220 K, HS)		
Intermolecular Contact	length	Length-VdW
N6···C15	3.211	-0.039
H11···S1	2.873	-0.127
H1···S2	2.782	-0.218
H1···C19	2.822	-0.078
H12B···N3	2.548	-0.202
H8A···S2	2.855	-0.145
H9···S2	2.959	-0.041
C1···S2	3.601	0.101
C7···C3	3.422	0.022

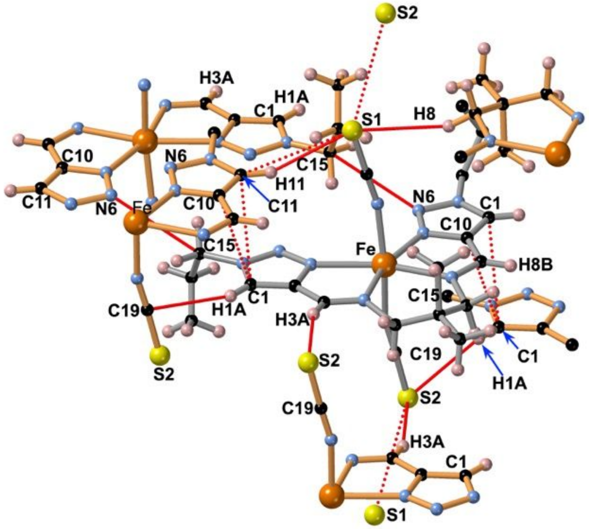

4 (300 K, HS)		
Intermolecular Contact	length	Length-VdW
H1A···S2	2.895	-0.105
H11···S1	2.826	-0.174
H3A···S2	2.999	-0.001
H8B···S1	2.974	-0.026
C11···S1	3.690	0.190
N6···C15	3.289	0.039

Figure S8. Intermolecular contacts for **4** (120 K, LS) (left), 220 K (HS) (middle) and 300 K (HS) (right). Solid and dotted lines represent, respectively, interatomic contacts shorter and slightly larger than the sum of VdW radii. Note that the column Length-VdW gives the difference between the actual interatomic length and the sum of the corresponding VdW radii.

Energy difference frameworks

Table S9. The full color-coded interaction mappings of a central reference molecule of **1** with the nearest neighbors and the contributions to the total energy in both spin states.

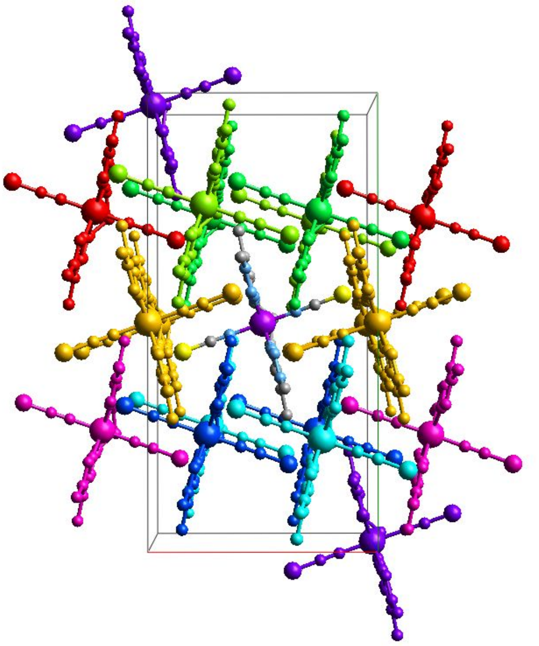

Color code	Spin state	Symmetry operation*	*R*, Å	Δ*R*(HS–LS), Å	Energy, kJ mol^{-1}					
					Electrostatic	Polarization	Dispersion	Exchange-repulsion	*E*(Total)	**Δ*E*(Total) (HS-LS)**
	LS<br>HS	x+1/4, -y+1/4, z+1/4	11.50<br>11.79	**0.29**	-24.9<br>-24.6	-6.1<br>-6.5	-7.2<br>-7.6	14.1<br>7.4	-28.3<br>-32.9	**-4.6**
	LS<br>HS	x+1/2, y, z+1/2	9.15<br>9.08	**-0.07**	-29.6<br>-21.4	-12.4<br>-12.4	-14.0<br>-12.8	16.0<br>10.7	-42.8<br>-36.4	**6.4**
	LS<br>HS	x+1/2, y, z+1/2	9.15<br>9.08	**-0.07**	-29.6<br>-23.7	-12.4<br>-13.9	-14.0<br>-14.6	16.0<br>15.4	-42.8<br>-38.5	**4.3**
	LS<br>HS	x+1/4, -y+1/4, z+1/4	12.28<br>12.23	**-0.05**	5.5<br>6.9	-1.5<br>-1.7	-6.2<br>-5.4	1.7<br>1.1	0.3<br>2.0	**1.7**
	LS<br>HS	x+3/4, -y+1/4, z+3/4	7.61<br>7.86	**0.25**	-55.2<br>-58.3	-41.6<br>-43.1	-80.9<br>-78.6	81.8<br>69.7	-109.1<br>-118.9	**-9.8**
	LS<br>HS	x+3/4, -y+3/4, z+1/4	12.30<br>12.22	**-0.08**	5.5<br>6.6	-1.2<br>-2.2	-4.3<br>-8.2	0.4<br>4.1	1.4<br>0.7	**-0.7**
	LS<br>HS	x+1/4, -y+3/4, z+3/4	7.64<br>7.84	**0.2**	-52.3<br>-61.4	-41.3<br>-43.4	-81.6<br>-77.6	79.8<br>71.5	-107.5<br>-120.4	**-12.9**
	LS<br>HS	x+1/2, y+1/2, z	13.63<br>14.24	**0.61**	8.6<br>10.8	-1.9<br>-2.2	-6.2<br>-5.6	5.1<br>3.9	5.4<br>7.3	**1.9**
	LS<br>HS	x+1/4, -y+3/4, z+3/4	11.52<br>11.78	**0.26**	-25.0<br>-24.4	-6.1<br>-6.4	-7.1<br>-7.7	14.1<br>7.4	-28.4<br>-32.7	**-4.3**

* corresponds to the crystal lattice in the LS state

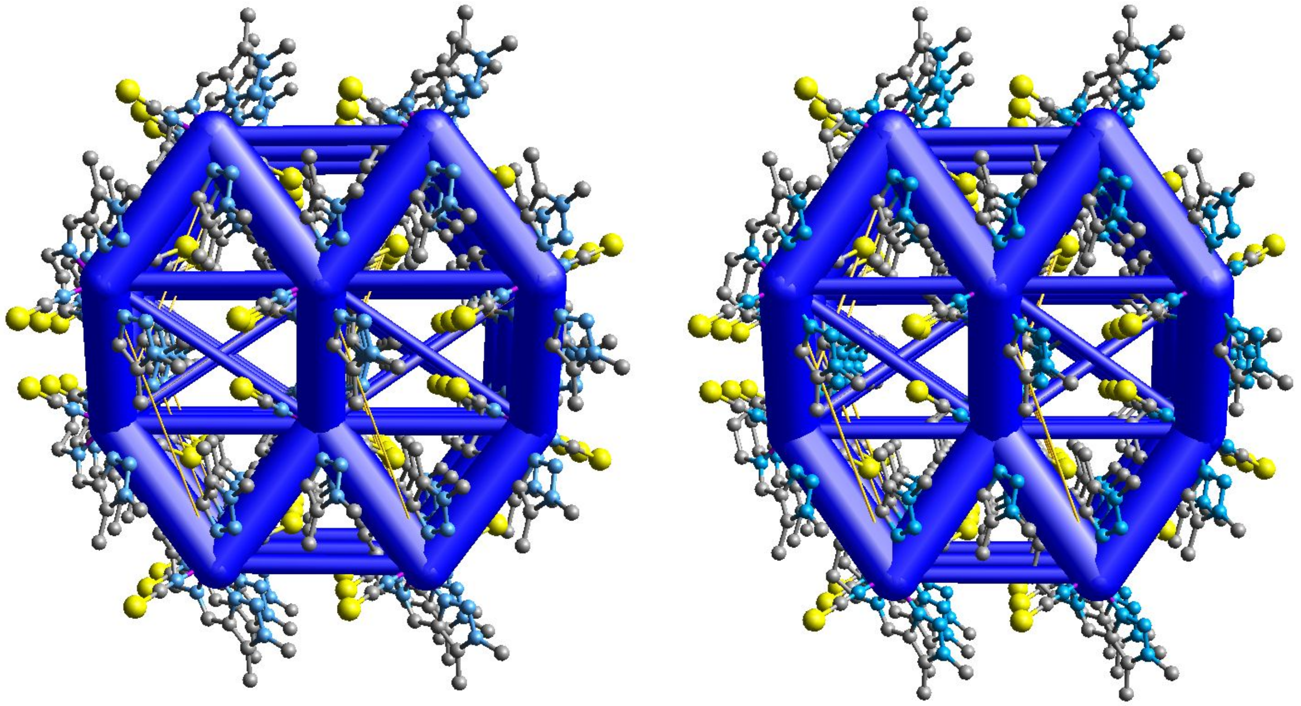

120K 250K

Figure S9. Energy framework of **1** constructed using ΔE(Total) values.

Table S10. The full color-coded interaction mappings of a central reference molecule of **2** with the nearest neighbors and the contributions to the total energy in both spin states.

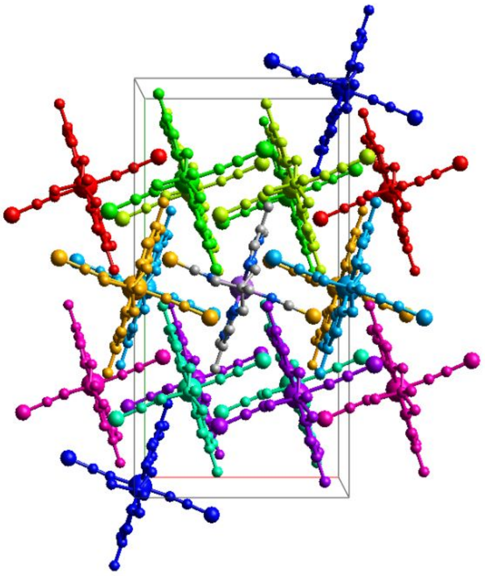

**Color code**	**Spin state**	**Symmetry operation***	***R*, Å**	**Δ*R*(HS–LS), Å**	**Energy, kJ mol^{-1}**					
					Electrostatic	Polarization	Dispersion	Exchange-repulsion	*E*(Total)	**Δ*E*(Total) (HS–LS)**
	LS<br>HS	x+1/4, -y+1/4, z+1/4	11.76<br>12.20	**0,44**	-24.2<br>-19.1	-6.1<br>-4.9	-7.9<br>-6.6	13.0<br>5.2	-29.0<br>-26.3	**2,7**
	LS<br>HS	x+1/2, y, z+1/2	9.78<br>9.12	**-0,66**	-22.0<br>-9.8	-10.7<br>-8.8	-15.0<br>-15.4	15.9<br>9.4	-34.4<br>-24.4	**10,0**
	LS<br>HS	x+3/4, -y+1/4, z+3/4	7.78<br>7.73	**-0,05**	-37.3<br>-58.6	-34.9<br>-34.2	-69.7<br>-77.1	54.1<br>62.4	-92.5<br>-115.9	**-23,4**
	LS<br>HS	x+1/4, -y+1/4, z+1/4	13.20<br>12.90	**-0,30**	2.2<br>3.2	-1.2<br>-1.6	-5.6<br>-8.3	1.5<br>4.7	-2.5<br>-2.1	**0,4**
	LS<br>HS	x+1/4, -y+3/4, z+3/4	7.78<br>8.30	**0,52**	-50.7<br>-47.0	-35.9<br>-33.9	-72.1<br>-62.6	72.5<br>53.8	-98.2<br>-96.0	**2,2**
	LS<br>HS	x+1/2, y, z+1/2	9.78<br>10.17	**0,39**	-22.3<br>-12.2	-11.3<br>-8.0	-14.9<br>-11.4	21.3<br>8.5	-31.7<br>-23.5	**8,2**
	LS<br>HS	x+1/2, y+1/2, z	13.62<br>14.26	**0,64**	8.8<br>9.9	-2.0<br>-2.5	-8.3<br>-8.1	8.1<br>10.5	5.7<br>8.0	**2,3**
	LS<br>HS	x+1/4, -y+3/4, z+3/4	13.20<br>13.33	**0,13**	2.1<br>1.2	-1.2<br>-1.4	-6.8<br>-8.0	3.1<br>4.4	-2.7<br>-4.1	**-1,4**
	LS<br>HS	x+1/4, -y+3/4, z+3/4	11.76<br>11.74	**-0,02**	-23.7<br>-23.8	-5.8<br>-6.5	-7.8<br>-7.6	13.0<br>11.8	-28.1<br>-29.4	**-1,3**

* corresponds to the crystal lattice in the LS state

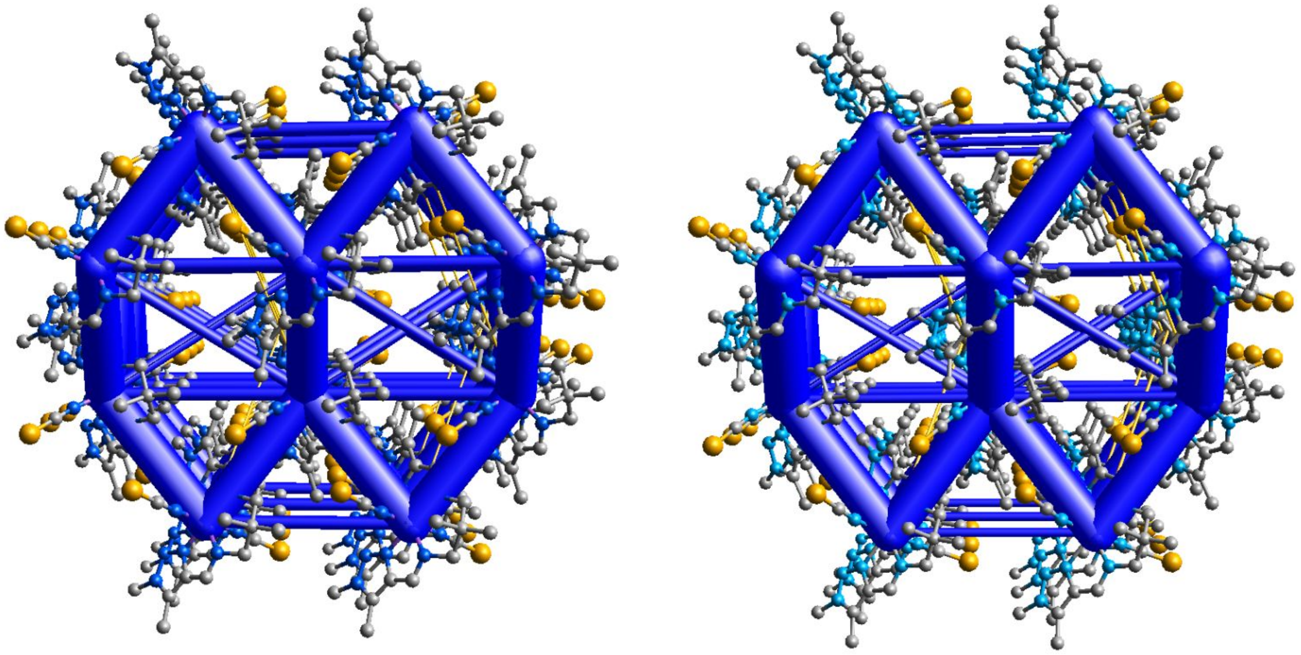

190 K 250 K

Figure S10. Energy framework of **2** constructed using ΔE(Total) values.

Table S11. The full color-coded interaction mappings of a central reference molecule of **4** with the nearest neighbors and the contributions to the total energy in both spin states.

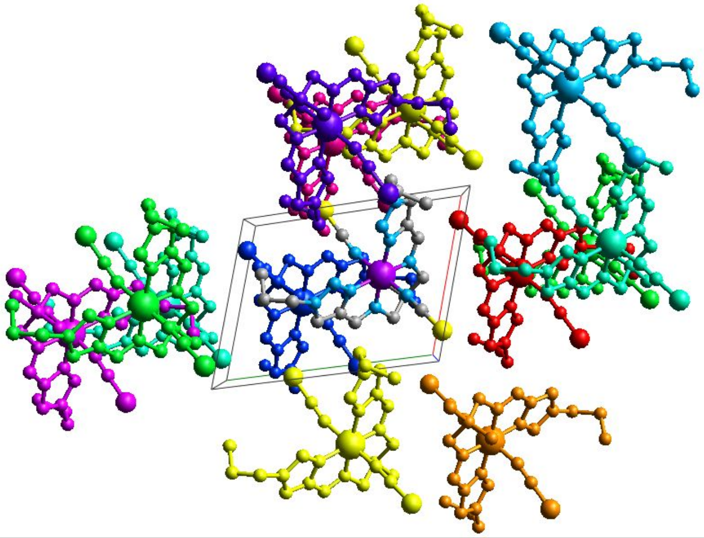

Color code	Spin state	Symmetry operation*	*R*, Å	Δ*R*(HS–LS), Å	Energy, kJ mol⁻¹: Electrostatic	Polarization	Dispersion	Exchange-repulsion	*E*(Total)	Δ*E*(Total) (HS–LS)
(red)	LS<br>HS	-x, -y, -z	7.99<br>8.03	**0.04**	-55.4<br>-56.4	-29.7<br>-27.4	-73.0<br>-68.0	80.5<br>67.6	-94.4<br>-97.4	**-3.0**
(orange)	LS<br>HS	-x, -y, -z	10.83<br>10.44	**-0.39**	-26.4<br>-19.7	-10.7<br>-9.7	-14.8<br>-12.8	14.2<br>7.9	-39.9<br>-34.2	**5.7**
(yellow)	LS<br>HS	x, y, z	8.52<br>8.92	**0.4**	-56.8<br>-61.9	-28.2<br>-32.1	-44.8<br>-50.4	46.0<br>53.9	-91.6<br>-99.9	**-8.3**
(yellow-green)	LS<br>HS	-x, -y, -z	7.77<br>7.96	**0.19**	-49.9<br>-32.7	-25.6<br>-20.3	-87.7<br>-69.2	88.2<br>62.5	-93.5<br>-71.2	**22.3**
(green)	LS<br>HS	x, y, z	14.29<br>14.51	**0.22**	3.8<br>2.1	-0.6<br>-0.6	-4.1<br>-6.3	1.0<br>2.4	0.6<br>-2.3	**-2.9**
(aquamarine)	LS<br>HS	x, y, z	12.02<br>12.47	**0.45**	-14.3<br>-15.0	-2.0<br>-2.1	-6.5<br>-4.6	2.9<br>0.8	-20.4<br>-21.0	**-0.6**
(sky blue)	LS<br>HS	-x, -y, -z	15.91<br>16.52	**0.61**	-2.6<br>-1.6	-0.2<br>-0.3	-1.6<br>-2.7	0.0<br>0.5	-4.3<br>-4.0	**0.3**
(blue)	LS<br>HS	-x, -y, -z	7.90<br>8.03	**0.13**	-53.9<br>-56.4	-25.8<br>-27.4	-75.2<br>-68.0	79.0<br>67.6	-92.7<br>-97.4	**-4.7**
(violet)	LS<br>HS	-x, -y, -z	8.79<br>8.54	**-0.25**	-28.3<br>-28.3	-20.8<br>-17.2	-83.6<br>-70.0	86.1<br>50.1	-64.9<br>-72.7	**-7.8**
(magenta)	LS<br>HS	-x, -y, -z	14.94<br>15.63	**0.69**	0.2<br>-2.6	-0.9<br>-0.9	-8.8<br>-8.4	4.4<br>4.2	-5.4<br>-8.1	**-2.7**
(pink)	LS<br>HS	-x, -y, -z	10.24<br>10.44	**0.2**	-1.1<br>-19.7	-7.5<br>-9.7	-10.0<br>-12.8	3.4<br>7.9	-13.4<br>-34.2	**-20.8**
(pink)	LS<br>HS	1+x,, 1+y, z	12.95<br>12.86	**-0.09**	21.3<br>25.6	-2.9<br>-3.7	-2.8<br>-3.3	2.5<br>6.0	19.5<br>25.1	**5.6**

* corresponds to the crystal lattice in the LS state

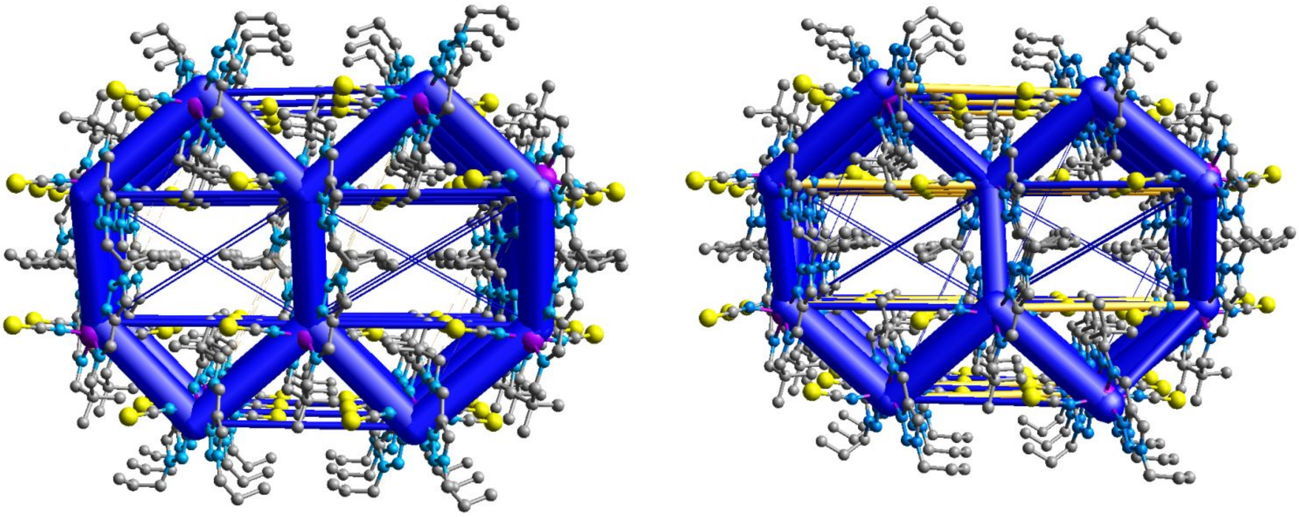

120K 220K

Figure S11. Energy framework of **4** constructed using Δ*E*(Total) value.

Table S12. The full color-coded interaction mappings of a central reference molecule of **Ph** with the nearest neighbors and the contributions to the total energy in both spin states.

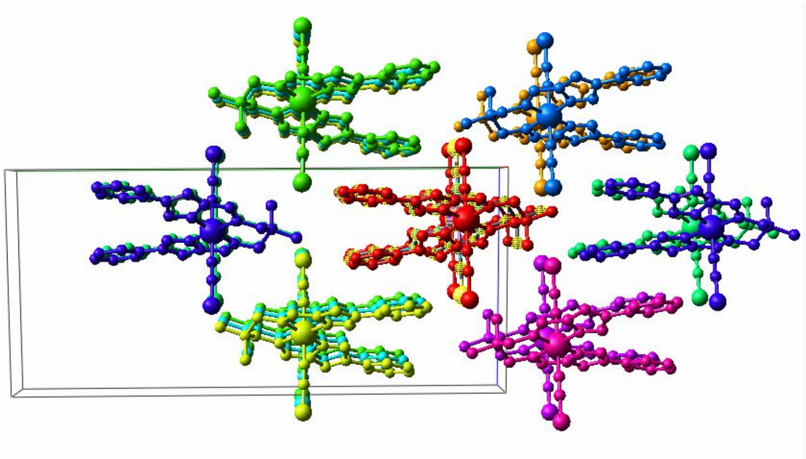

Color code	Spin state	Symmetry operation*	*R*, Å	Δ*R*(HS–LS), Å	Energy, kJ mol⁻¹					
					Electrostatic	Polarization	Dispersion	Exchange-repulsion	*E*(Total)	**Δ*E*(Total) (HS–LS)**
	LS<br>HS	x, y, z	7,17<br>8,16	**0,99**	-65,5<br>-71,8	-33,7<br>-33,1	-106,3<br>-73,5	95,0<br>77,6	-128,1<br>-116,5	**11,6**
	LS<br>HS	-x, -y, -z	11,40<br>11,58	**0,18**	-32,2<br>-19,3	-13,4<br>-8,6	-19,0<br>-9,9	29,6<br>6,1	-42,2<br>-31,6	**10,6**
	LS<br>HS	x+1/2, -y+1/2, z+1/2	9,53<br>9,04	**-0,49**	-25,0<br>-22,4	-8,5<br>-11,4	-12,6<br>-13,9	9,8<br>13,8	-37,6<br>-35,7	**1,9**
	LS<br>HS	x+1/2, -y+1/2, z+1/2	9,53<br>10,04	**0,51**	-25,0<br>-1,6	-8,5<br>-3,8	-12,6<br>-11,1	9,8<br>0,4	-37,6<br>-13,9	**23,7**
	LS<br>HS	-x+1/2, y+1/2, -z+1/2	14,72<br>14,13	**-0,59**	2,8<br>4,6	-0,6<br>-0,4	-10,0<br>-4,6	6,3<br>1,5	-2,4<br>1,4	**3,8**
	LS<br>HS	x+1/2, -y+1/2, z+1/2	15,35<br>15,61	**0,26**	6,0<br>4,6	-1,8<br>-0,8	-11,9<br>-22,4	7,9<br>11,1	-0,4<br>-8,4	**-8,0**
	LS<br>HS	x+1/2, -y+1/2, z+1/2	15,35<br>17,86	**2,51**	6,0<br>5,4	-1,8<br>-0,8	-11,9<br>-4,4	7,9<br>1,0	-0,4<br>1,9	**2,3**
	LS<br>HS	x+1/2, -y+1/2, z+1/2	10,57<br>10,28	**-0,29**	-27,1<br>-28,4	-14,2<br>-11,7	-62,9<br>-69,7	47,7<br>49,8	-64,5<br>-68,6	**-4,1**
	LS<br>HS	x+1/2, -y+1/2, z+1/2	10,57<br>11,57	**1,00**	-27,1<br>-19,3	-14,2<br>-11,5	-62,9<br>-56,1	47,7<br>27,4	-64,5<br>-60,8	**3,7**
	LS<br>HS	-x, -y, -z	10,22<br>9,80	**-0,42**	-35,4<br>-50,4	-22,3<br>-28,3	-39,9<br>-43,5	40,7<br>56,4	-63,5<br>-77,2	**-13,7**
	LS<br>HS	-x+1/2, y+1/2, -z+1/2	14,89<br>14,95	**0,06**	2,0<br>2,7	-0,8<br>-0,7	-12,0<br>-7,4	8,2<br>1,8	-3,8<br>-3,0	**0,8**
	LS<br>HS	-x, -y, -z	10,38<br>9,80	**-0,58**	-44,1<br>-50,4	-17,6<br>-28,3	-27,2<br>-43,5	34,7<br>56,4	-61,9<br>-77,2	**-15,3**
	LS<br>HS	-x, -y, -z	11,96<br>11,58	**-0,38**	-9,2<br>-19,3	-6,9<br>-8,6	-9,9<br>-9,9	11,5<br>6,1	-16,3<br>-31,6	**-15,3**

* corresponds to the crystal lattice in the LS state

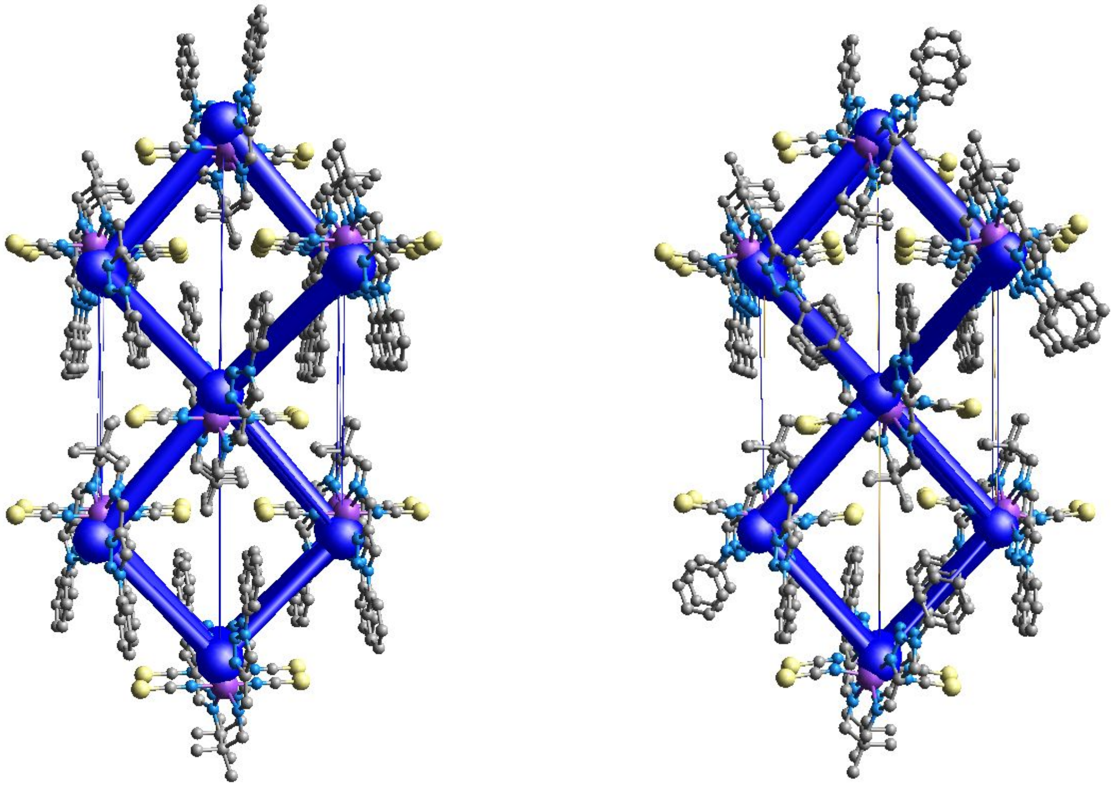

150K 448K

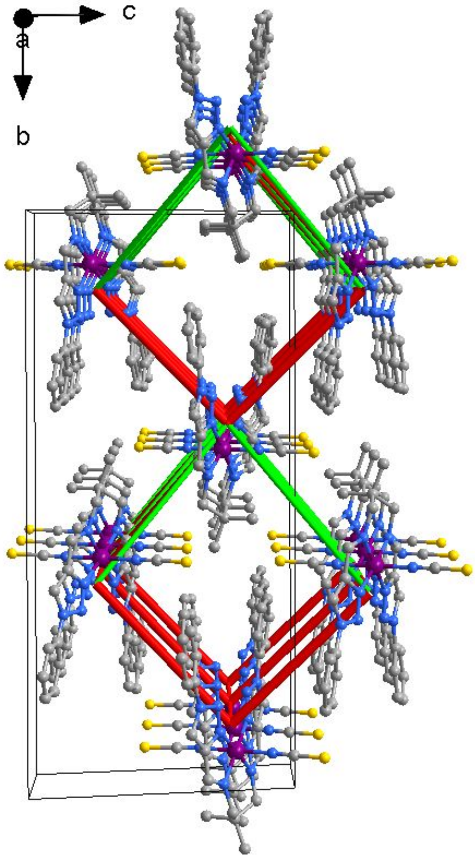

Figure S12. Above are energy framework of **Ph** ploted using Δ*E*(Total) values. Below is energy difference framework of **Ph** constructed using Δ*E*(Total)(HS-LS) values. Tube size is scaled proportionally to the absolute value of the interaction energy, cut-off is 5 kJ mol^{-1}.

Table S13. The full color-coded interaction mappings of a central reference molecule of **Tol** with the nearest neighbors and the contributions to the total energy in both spin states.

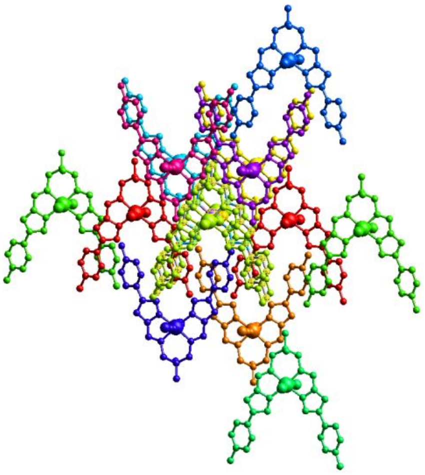

Color code	Spin state	Symmetry operation*	*R*, Å	Δ*R*(HS–LS), Å	Energy, kJ mol⁻¹ Electrostatic	Polarization	Dispersion	Exchange-repulsion	*E*(Total)	Δ*E*(Total) (HS–LS)
(red)	LS<br>HS	x, y, z	7.71<br>8.26	**0,55**	-72.5<br>-70.1	-31.0<br>-31.4	-64.5<br>-60.7	84.0<br>65.2	-103.9<br>-110.0	**-6,1**
(orange)	LS<br>HS	-x, -y, -z	8.86<br>8.80	**-0,06**	-31.1<br>-22.3	-16.1<br>-15.1	-107. 3<br>-92.1	88.8<br>52.1	-83.4<br>-82.7	**0,7**
(yellow)	LS<br>HS	-x, -y, -z	10.97<br>11.54	**0,57**	-30.1<br>-25.8	-12.4<br>-11.4	-20.5<br>-15.2	32.9<br>16.0	-38.5<br>-39.1	**-0,6**
(yellow-green)	LS<br>HS	x, y, z	12.56<br>12.84	**0,28**	22.6<br>26.8	-3.9<br>-4.6	-3.9<br>-3.8	10.3<br>10.7	24.0<br>28.2	**4,2**
(green)	LS<br>HS	x, y, z	15.96<br>17.22	**1,26**	2.9<br>3.4	-0.7<br>-0.8	-6.6<br>-6.6	3.2<br>3.9	-1.2<br>-0.4	**0,8**
(green)	LS<br>HS	x, y, z	17.42<br>17.26	**-0,16**	1.4<br>1.4	-0.2<br>-0.4	-5.1<br>-7.1	2.0<br>4.7	-1.9<br>-2.0	**-0,1**
(green)	LS<br>HS	x, y, z	17.42<br>17.18	**-0,24**	1.4<br>1.2	-0.2<br>-0.3	-5.1<br>-7.1	2.0<br>3.6	-1.9<br>-3.0	**-1,1**
(aquamarine)	LS<br>HS	-x, -y, -z	15.48<br>16.44	**0,96**	-3.1<br>-2.3	-2.0<br>-1.6	-15.9<br>-11.0	6.3<br>2.1	-14.6<br>-11.9	**2,7**
(sky blue)	LS<br>HS	-x, -y, -z	9.58<br>9.89	**0,31**	-42.9<br>-48.1	-25.1<br>-27.5	-60.1<br>-50.4	66.3<br>50.3	-75.3<br>-84.0	**-8,7**
(blue)	LS<br>HS	x, y, z	17.21<br>17.18	**-0,03**	0.6<br>1.2	-0.3<br>-0.3	-6.7<br>-7.1	4.2<br>3.6	-2.9<br>-3.0	**-0,1**
(dark blue)	LS<br>HS	-x, -y, -z	8.27<br>8.10	**-0,17**	-28.9<br>-21.4	-19.2<br>-15.9	-126.5<br>-104.6	104.8<br>59.9	-90.2<br>-88.5	**1,7**
(violet)	LS<br>HS	-x, -y, -z	10.04<br>9.89	**-0,15**	-53.8<br>-48.1	-28.0<br>-27.5	-53.3<br>-50.4	72.1<br>50.3	-79.4<br>-84.0	**-4,6**
(magenta)	LS<br>HS	-x, -y, -z	15.80<br>16.23	**0,43**	-2.0<br>-1.7	-1.7<br>-1.6	-12.3<br>-8.4	4.8<br>1.2	-11.1<br>-9.5	**1,6**
(pink-red)	LS<br>HS	-x, -y, -z	11.93<br>11.54	**-0,39**	-18.0<br>-25.8	-9.7<br>-11.4	-13.2<br>-15.2	16.0<br>16.0	-27.8<br>-39.1	**-11,3**

* corresponds to the crystal lattice in the LS state

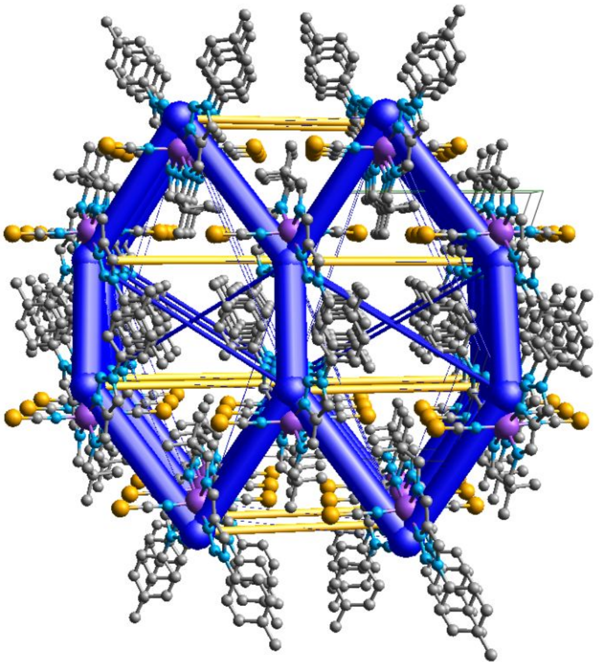

220K

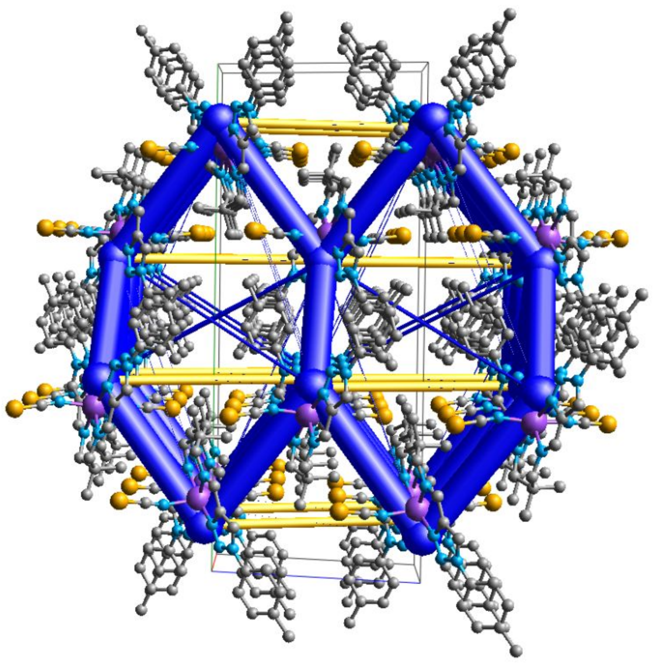

250K

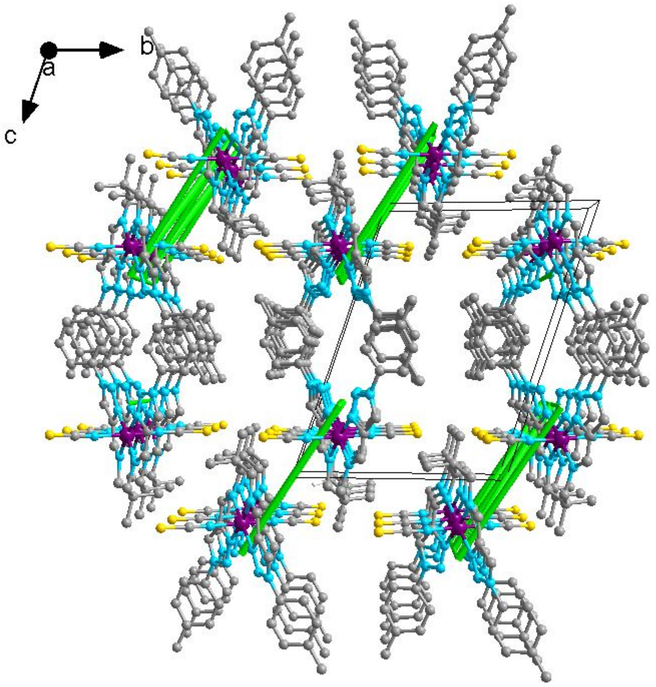

Figure S13. Above are energy frameworks of **Tol** constructed using ΔE(Total) values. Below is energy difference framework of **Tol** constructed using ΔE(Total)(HS-LS) values (below). Tube size is scaled proportionally to the absolute value of the interaction energy, cut-off is 5 kJ mol^{-1}.

Table S14. The full color-coded interaction mappings of a central reference molecule of **MeA** with the nearest neighbors and the contributions to the total energy in both spin states.

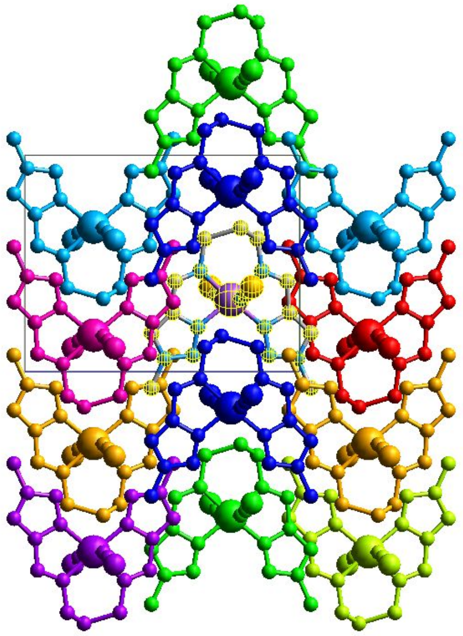

Color code	Spin state	Symmetry operation*	***R*, Å**	**Δ*R*(HS–LS), Å**	Energy, kJ mol^{-1}					
					Electrostatic	Polarization	Dispersion	Exchange-repulsion	*E*(Total)	**Δ*E*(Total) (HS-LS)**
	LS<br>HS	-x+1/2, -y+1/2, -z	8.24<br>9.29	**1,05**	-68.7<br>-63.1	-29.7<br>-27.9	-42.4<br>-34.3	58.3<br>47.2	-95.5<br>-88.0	**7,5**
	LS<br>HS	x, -y, z+1/2	8.89<br>7.53	**-1,36**	-40.6<br>-52.9	-18.1<br>-29.1	-41.2<br>-52.3	27.3<br>50.8	-75.3<br>-91.7	**-16,4**
	LS<br>HS	-x+1/2, -y+1/2, -z	13.55<br>13.88	**0,33**	1.6<br>-0.9	-0.6<br>-0.6	-1.4<br>-1.4	0.1<br>0.1	0.0<br>-2.5	**-2,5**
	LS<br>HS	x, y, z	9.79<br>8.93	**-0,86**	10.5<br>10.3	-2.5<br>-4.9	-16.4<br>-20.7	7.4<br>9.1	-0.5<br>-5.2	**-4,7**
	LS<br>HS	-x+1/2, y+1/2, -z+1/2	9.09<br>9.34	**0,25**	-22.0<br>-15.5	-14.3<br>-14.1	-17.3<br>-16.2	23.9<br>21.3	-34.1<br>-27.7	**6,4**
	LS<br>HS	x, -y, z+1/2	7.68<br>9.12	**1,44**	-44.3<br>-48.9	-24.5<br>-14.1	-41.7<br>-30.2	56.6<br>22.9	-66.4<br>-74.3	**-7,9**
	LS<br>HS	-x+1/2, y+1/2, -z+1/2	9.04<br>9.27	**0,23**	-20.9<br>-14.3	-14.9<br>-14.9	-18.8<br>-18.2	26.9<br>22.1	-32.9<br>-28.4	**4,5**
	LS<br>HS	-x+1/2, -y+1/2, -z	13.56<br>13.87	**0,31**	1.6<br>-0.7	-0.6<br>-0.6	-1.4<br>-1.4	0.1<br>0.1	0.1<br>-2.4	**-2,5**
	LS<br>HS	-x+1/2, -y+1/2, -z	8.24<br>9.27	**1,03**	-67.3<br>-61.8	-29.7<br>-27.6	-42.8<br>-34.7	58.7<br>47.3	-94.1<br>-86.8	**7,3**

* corresponds to the crystal lattice in the LS state

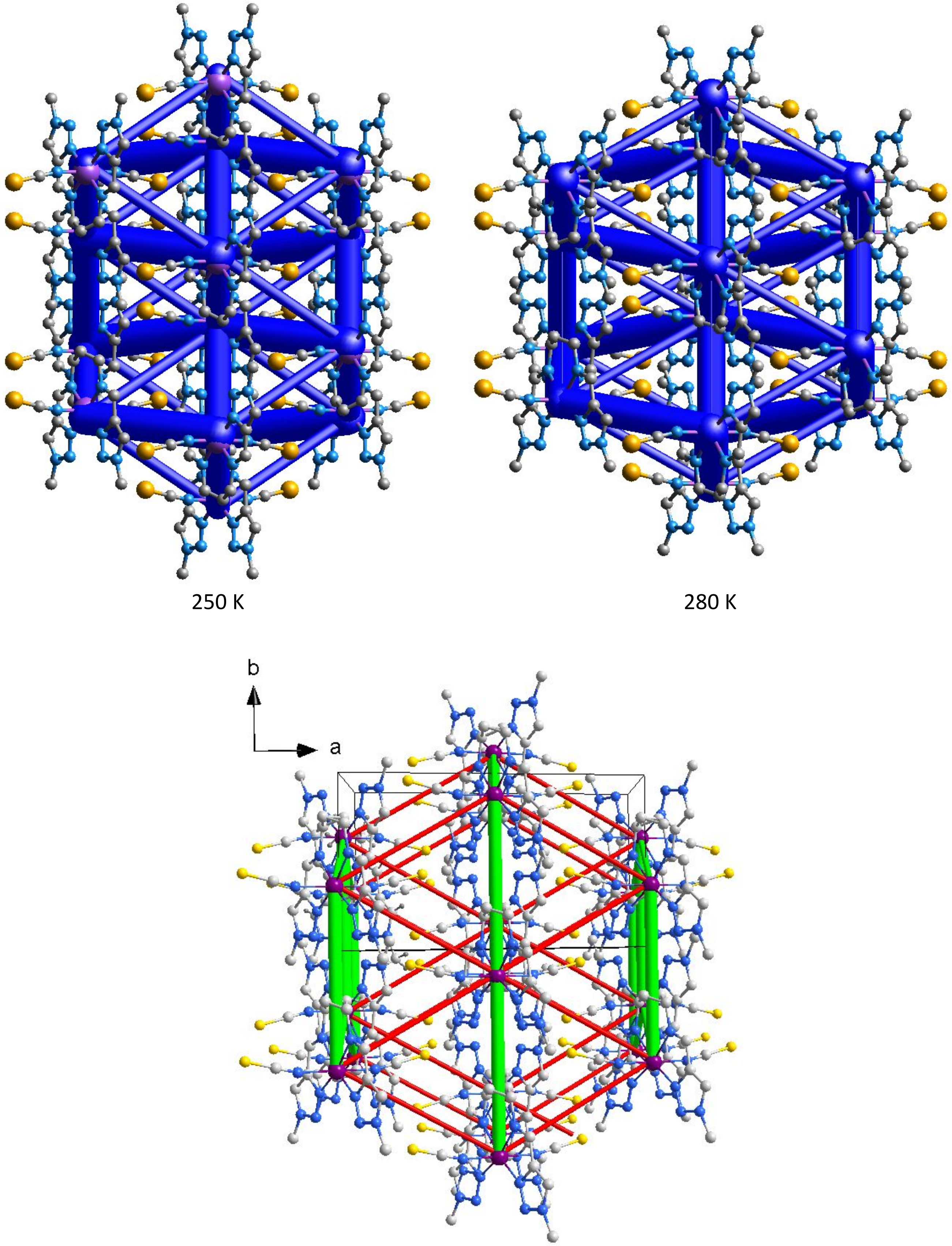

Figure S14. Above are energy frameworks of **MeA** constructed using Δ*E*(Total) values. Below is energy difference framework of **MeA** constructed using Δ*E*(Total)(HS-LS) values. Tube size is scaled proportionally to the absolute value of the interaction energy, cut-off is 5 kJ mol^{-1}.

Table S15. The full color-coded interaction mappings of a central reference molecule of **MeB** with the nearest neighbors and the contributions to the total energy in both spin states.

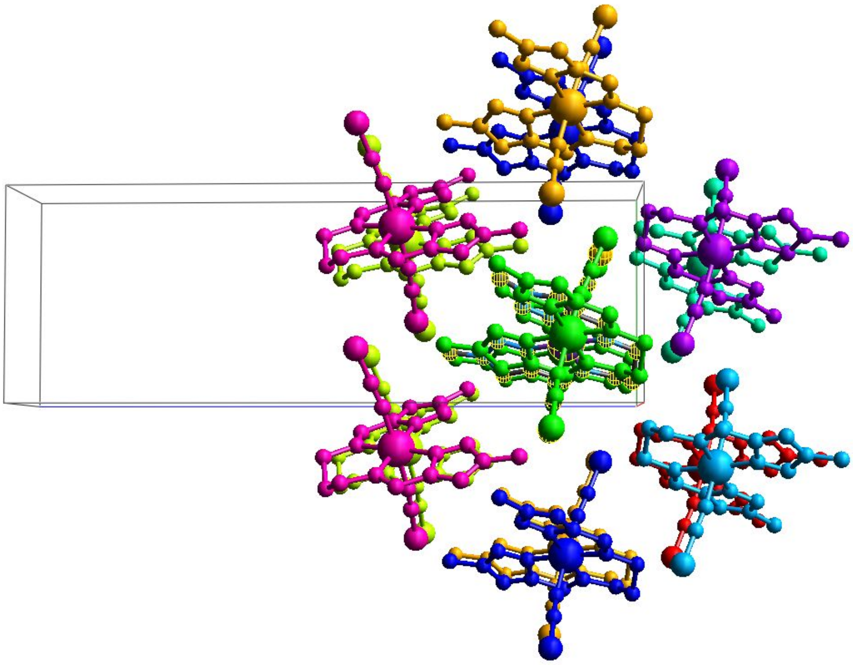

Color code	Spin state	Symmetry operation*	*R*, Å	Δ*R*(HS–LS), Å	Energy, kJ mol^{-1}					
					Electrostatic	Polarization	Dispersion	Exchange-repulsion	*E*(Total)	**Δ*E*(Total) (HS-LS)**
red	LS<br>HS	-x, -y, -z	10.76<br>11.15	**0,39**	10.2<br>10.1	-1.6<br>-1.2	-8.8<br>-5.2	3.6<br>0.6	4.2<br>5.7	**1,5**
orange	LS<br>HS	x, y, z	12.11<br>12.58	**0,47**	3.3<br>3.5	-1.1<br>-1.3	-2.1<br>-2.4	0.0<br>0.0	0.9<br>0.6	**-0,3**
yellow-green	LS<br>HS	-x+1/2, y+1/2, -z+1/2	8.30<br>8.61	**0,31**	-55.8<br>-48.1	-19.3<br>-15.7	-41.2<br>-33.3	45.4<br>28.4	-81.1<br>-73.9	**7,2**
green	LS<br>HS	x, y, z	8.13<br>8.50	**0,37**	-52.8<br>-64.6	-24.8<br>-27.4	-38.0<br>-40.4	36.0<br>38.8	-85.0<br>-99.8	**-14,8**
aquamarine	LS<br>HS	-x, -y, -z	9.99<br>9.82	**-0,17**	-37.3<br>-35.2	-12.3<br>-11.3	-14.7<br>-12.4	31.8<br>20.4	-41.7<br>-43.8	**-2,1**
light blue	LS<br>HS	-x, -y, -z	8.90<br>9.33	**0,43**	-18.7<br>-26.7	-11.9<br>-13.3	-29.6<br>-30.8	20.3<br>36.2	-41.7<br>-42.5	**-0,8**
blue	LS<br>HS	x, y, z	8.97<br>9.27	**0,3**	-33.9<br>-34.9	-15.7<br>-15.0	-19.7<br>-17.2	22.8<br>19.2	-50.6<br>-51.0	**-0,4**
violet	LS<br>HS	-x, -y, -z	7.95<br>7.70	**-0,25**	-33.8<br>-27.7	-21.7<br>-23.6	-36.4<br>-38.0	38.5<br>32.8	-59.7<br>-59.5	**0,2**
magenta	LS<br>HS	-x+1/2, y+1/2, -z+1/2	9.16<br>9.02	**-0,14**	-8.5<br>-2.8	-9.4<br>-7.6	-22.9<br>-18.7	13.2<br>8.9	-27.8<br>-19.3	**8,5**

* relates to the crystal lattice in the LS state

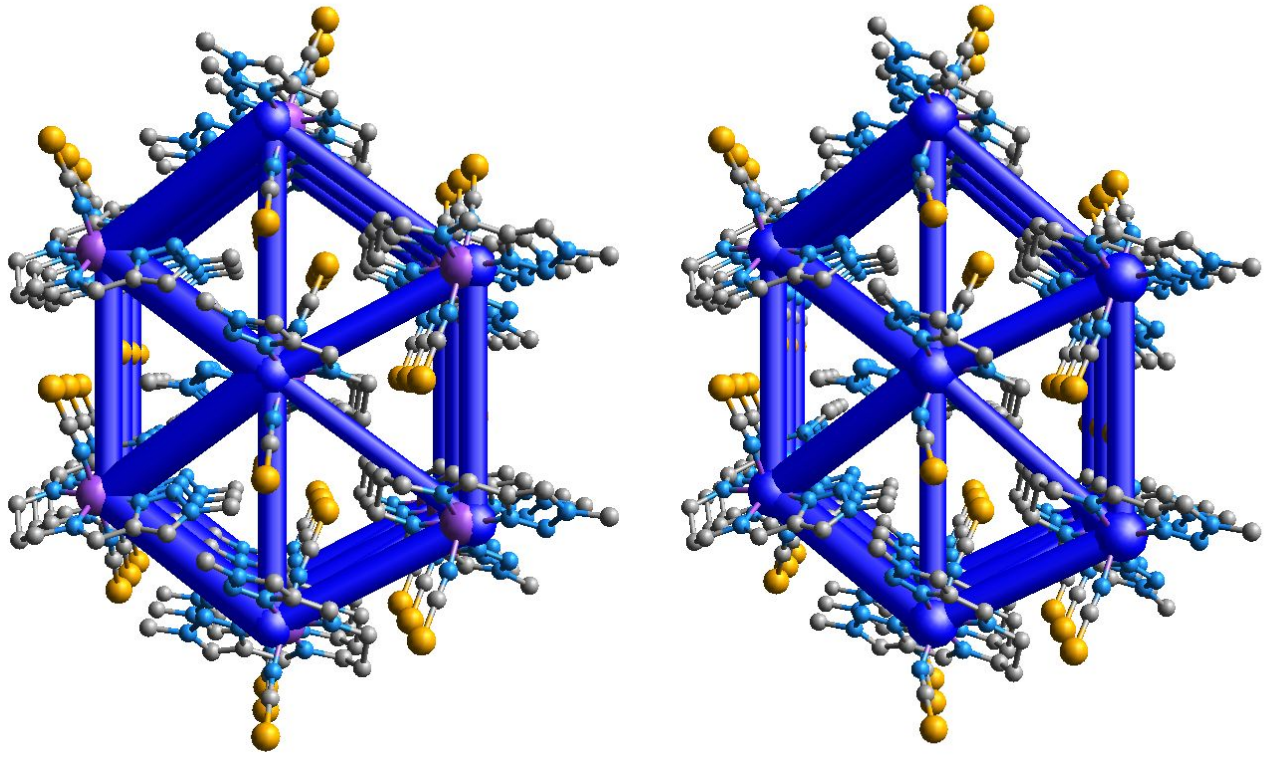

298 K 380 K

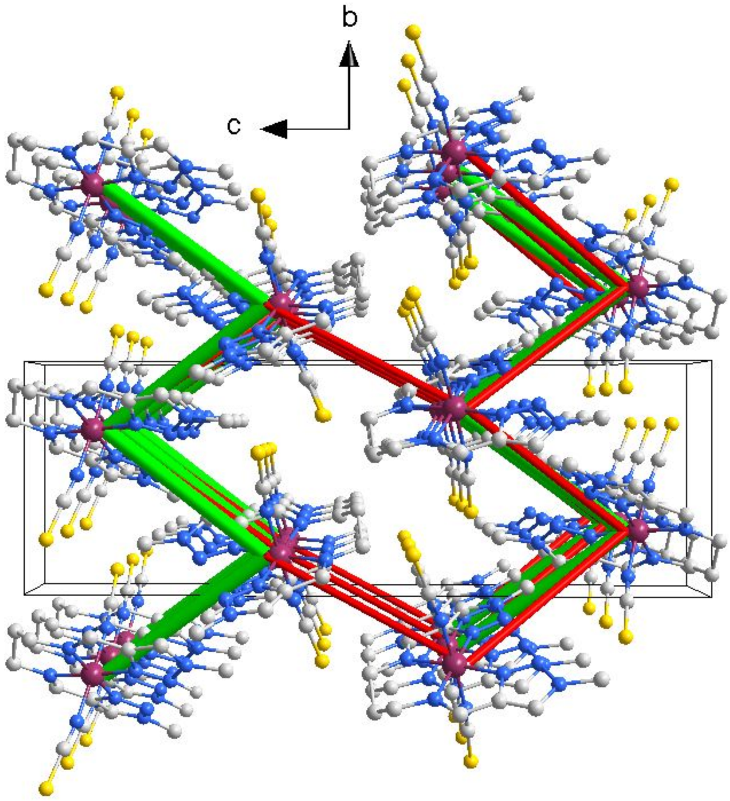

Figure S15. Energy framework of **MeB** constructed using Δ*E*(Total) values (above). Energy difference framework of **MeB** constructed using Δ*E*(Total)(HS-LS) values (below). Tube size is scaled proportionally to the absolute value of the interaction energy, cut-off is 5 kJ mol^{-1}.

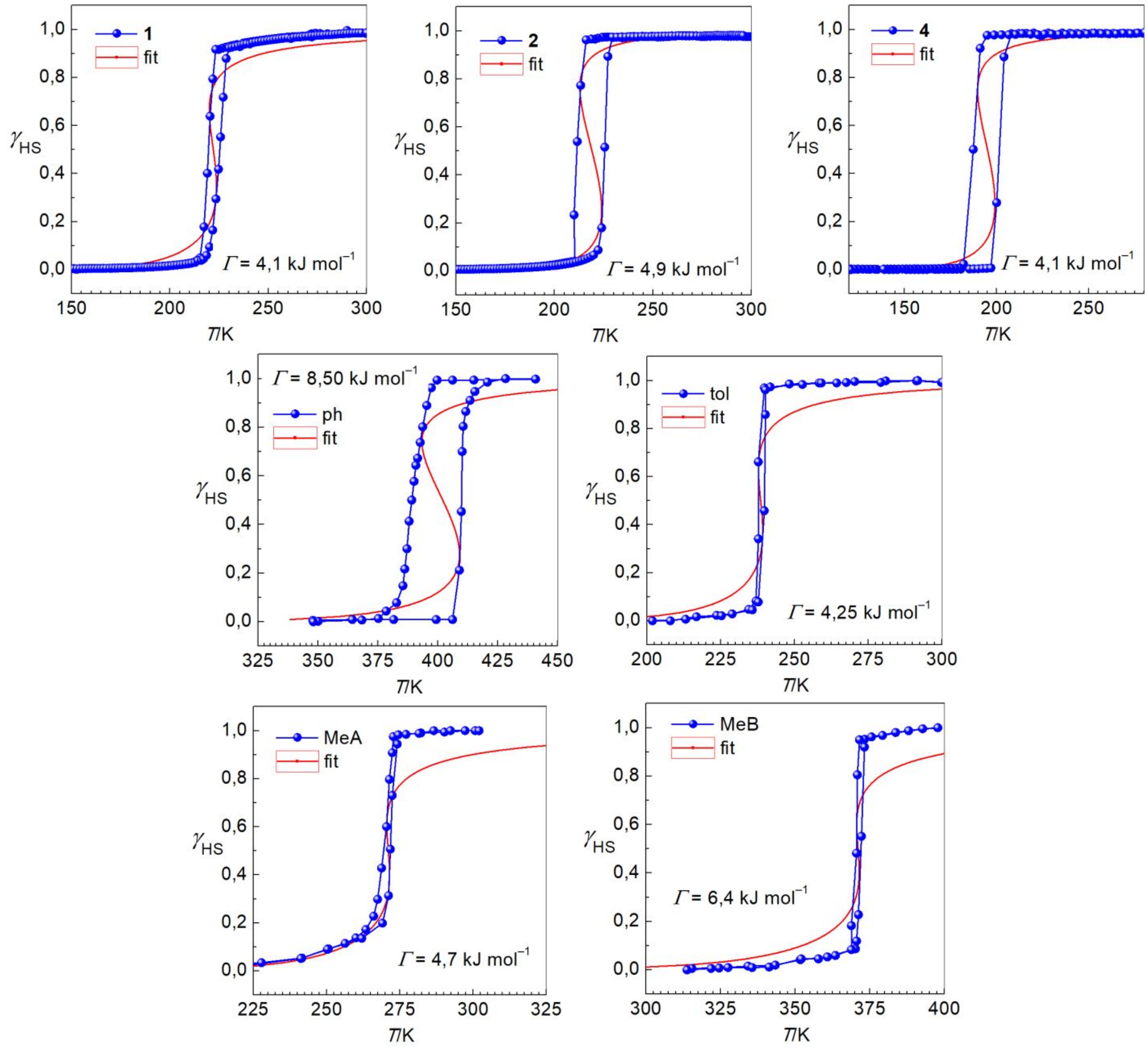

Figure S16. Slichter-Drickamer model fitting of the magnetic hysteresis loops γ_{HS} vs T of the title and literature compounds. The loops for **Ph**, **Tol**, **MeA** and **MeB** were constructed using data from refs. 1,2.

References

(1) Hagiwara, H.; Masuda, T.; Ohno, T.; Suzuki, M.; Udagawa, T.; Murai, K.-i. Neutral Molecular Iron(II) Complexes Showing Tunable Bistability at Above, Below, and Just Room Temperature by a Crystal Engineering Approach: Ligand Mobility into a Three-Dimensional Flexible Supramolecular Network. *Cryst. Growth Des.* **2017**, *17* (11), 6006-6019.

(2) Hagiwara, H.; Okada, S. A polymorphism-dependent $T_{1/2}$ shift of 100 K in a hysteretic spin-crossover complex related to differences in intermolecular weak CH•••X hydrogen bonds (X = S *vs.* S and N). *Chem. Commun.* **2016**, *52* (4), 815-818.
